# *Ectophoma salviniae* sp. nov., *Neottiosporina mihintaleensis* sp. nov. and four other endophytes associated with aquatic plants from Sri Lanka and their extracellular enzymatic potential

**DOI:** 10.3389/fcimb.2024.1475114

**Published:** 2025-01-08

**Authors:** Madhara K. Wimalasena, Nalin N. Wijayawardene, Thushara C. Bamunuarachchige, Gui-Qing Zhang, R. G. Udeni Jayalal, Darbhe J. Bhat, Turki M. Dawoud, Heethaka K. S. de Zoysa, Dong-Qin Dai

**Affiliations:** ^1^ Center for Yunnan Plateau Biological Resources Protection and Utilization, Qujing Normal University, Qujing, Yunnan, China; ^2^ Faculty of Graduate Studies, Sabaragamuwa University of Sri Lanka, Belihuloya, Sri Lanka; ^3^ Department of Bioprocess Technology, Faculty of Technology, Rajarata University of Sri Lanka, Mihintale, Sri Lanka; ^4^ Tropical Microbiology Research Foundation, Colombo, Sri Lanka; ^5^ Department of Natural Resources, Faculty of Applied Sciences, Sabaragamuwa University of Sri Lanka, Belihuloya, Sri Lanka; ^6^ Department of Botany and Microbiology, College of Science, King Saud University, Riyadh, Saudi Arabia; ^7^ Vishnugupta Vishwavidyapeetam, Gokarna, India

**Keywords:** freshwater plants, fungal endophytes, hydrolytic and oxidative enzymes, new species, phylogeny, taxonomy

## Abstract

Endophytic fungi associated with selected aquatic plants, *Eichhornia crassipes, Nymphaea nouchali, Salvinia minima* and *S. molesta* were evaluated. *Ectophoma salviniae* sp. nov. and *Neottiosporina mihintaleensis* sp. nov. are introduced as novel taxa from *Salvinia* spp. from Sri Lanka. *Chaetomella raphigera* is reported as a new geographical record, *Colletotrichum siamense* and *C. truncatum* are reported as novel host records in aquatic plants, while *Phyllosticta capitalensis* has been identified on the same host (*Nymphaea nouchali*) in the North-Central Province of Sri Lanka. Identification of the fungi was based on morphological characteristics and multi-locus phylogenetic analyses using ITS, LSU, SSU, *ACT*, *CHS-*1, *GAPDH*, *tub*2, *rpb*2, and *tef*1-α molecular markers. The identified fungi were analysed for extracellular enzymatic properties. According to the qualitative analysis, *Ectophoma salviniae* sp. nov. exhibited the highest amylase production, *Chaetomella raphigera* exhibited the highest cellulase enzyme production, and *Neottiosporina mihintaleensis* sp. nov. exhibited the highest laccase production. The results demonstrate the aquatic fungal diversity in this region and their extracellular enzymatic potentials, providing valuable insights for future biotechnological approaches.

## 1 Introduction

The magnitude of the fungal kingdom has been a debatable topic for decades and several studies have revised the prevailing estimations based on the advancements in estimation methods and technologies (e.g., Hawksworth, 1991, 2001; May, 2000; Tedersoo et al., 2015, 2022; Hyde et al., 2023). However, Wu et al. (2019) estimated the global fungal species diversity to be around 12 million, based on culture-independent approaches, while culture-dependent methods yielded a more conservative estimate of 2.2 to 3.8 million species (Hawksworth and Lücking, 2017). Niskanen et al. (2023) revisited the species number estimated by Hawksworth and Lücking (2017) and concluded that it would be 2–3 million species, however, the best estimation is at 2.5 million. Nevertheless, only 160,000 fungal species have been accepted in Species Fungorum (2024; accession date: 06 June 2024, https://www.speciesfungorum.org/names/names.asp), thus, a large number of taxa are yet to be described. Additionally, it has reported understudied geographical regions and well-studied hosts but biodiversity regions (temperate and tropical) would harbour more novel taxa (Wijayawardene et al., 2021). It is considered a challenge to reveal the unknown fungal diversity with traditional methods, such as morphological or cultural characteristics. The recent advances in molecular techniques (such as high-throughput sequencing of environmental samples) are accelerating the explorations and further trying to reveal the understudied fungal habitats, life modes and geographical regions (Wijayawardene et al., 2023).

Endophytes are widespread and have been reported in plants from diverse ecosystems such as deserts, temperate zones, arctic tundra, tropical forests, grasslands, and croplands (Arnold, 2007; Arnold and Lutzoni, 2007; Zheng et al., 2015; Rana et al., 2019; Harrison and Griffin, 2020; Dar et al., 2022; Hashem et al., 2023). Based on the ratio of the host (vascular plants) to species current estimates suggest that there are approximately one million species of fungal endophytes (Sun and Guo, 2012; Lugtenberg et al., 2016; Rashmi et al., 2019; Wu et al., 2019; Bhunjun et al., 2024). Endophytic fungi belong to both mitosporic and meiosporic ascomycetes, which reside within plants without causing symptoms and colonize healthy tissue beneath the epidermal cell layer through quiet infections (Lu et al., 2012; Ali et al., 2018; Abdel-Wareth, 2022). Further, it has been reported that comparing endophytic basidiomycetes and basal fungi with endophytic ascomycetes shows that almost 90% of the identified endophytes are *Ascomycota* (Rungjindamai and Gareth Jones, 2024).

Endophytes’ ecology, evolution, and applications are interesting topics; however, knowledge about their diversity, geographic and ecological distributions in most plant communities remains limited and unexplored (Gao et al., 2019; Zheng et al., 2021). Previous research has primarily focused on endophytes in terrestrial plants, while endophytic fungal studies related to aquatic plants received little attention (Li et al., 2010; Sandberg et al., 2014; Dissanayake et al., 2016; Myovela et al., 2024). However, the richness of endophytic fungal diversity has been reported in marine ecosystems (Kamat et al., 2020; El-Bondkly et al., 2021), mangrove ecosystems (Deshmukh et al., 2020; Jia et al., 2020; da Silveira Bastos et al., 2024; Myovela et al., 2024), and freshwater ecosystems (You et al., 2015; Chen et al., 2023; Pramanic et al., 2023). Aquatic plants (including emergent plants, floating-leaved plants, free-floating plants, submerged plants, and wet plants (Ismail et al., 2021; Zhou et al., 2023) serve as hosts for a diverse array of endophytic fungi (Zheng et al., 2021; Wimalasena et al., 2024). Many researchers have studied the diversity and ecological roles of aquatic plants (O’Hare et al., 2018; Kamat et al., 2020; Zheng et al., 2022; Ji et al., 2024). However, most studies have overlooked how endophytes affect these plants and their wider ecological functions. Accordingly, there is a significant gap in the understanding of endophytic fungal communities in aquatic plants (Sandberg et al., 2014; Zheng et al., 2021; Wimalasena et al., 2024).

Sri Lanka is a tropical biodiversity hotspot (Gunawardene et al., 2007; Surasinghe et al., 2019; Sarathchandra et al., 2021; De Zoysa, 2022) and harbours a diverse range of aquatic ecosystems, including both coastal and inland areas (Gunatilleke et al., 2008). Inland freshwater habitats (rivers, streams, marshes, swamp forests, villus, and man-made reservoirs) collectively cover approximately 202,435 hectares (Gunatilleke et al., 2008; Yakandawala, 2012). Sri Lanka is home to over 370 species of aquatic and wetland plants, with 12% being endemic to the country (Yakandawala, 2012). These plants serve as habitats for various fungi, including endophytes (Ratnaweera, 2019; Wimalasena et al., 2024). As highlighted by Wimalasena et al. (2024), Sri Lanka offers significant potential for the identification and study of endophytic fungi. Previous research on endophytic fungi in freshwater plants has been relatively limited in Sri Lanka. For instance, Dissanayake et al. (2016) isolated 20 distinct endophytic fungi from *Nymphaea nouchali*. More recently, Wimalasena et al. (2024) reported the ongoing study on the isolation of endophytic fungi from freshwater plants in Sri Lanka.

In this study, an effort was made to document the endophytic fungi associated with three freshwater plant taxa, *viz*., *Eichhornia crassipes, Nymphaea nouchali*, *Salvinia minima* and *S. molesta*, found in the lentic freshwater habitats of the Mihintale area (in Anuradhapura district, North-Central Province), Sri Lanka. We isolated six fungal species that belong to *Colletotrichum*, *Chaetomella*, *Ectophoma*, *Neottiosporina* and *Phyllosticta*. Among these taxa, two new species, *Ectophoma salviniae* sp. nov. and *Neottiosporina mihintaleensis* sp. nov. are introduced. *Chaetomella raphigera* has been reported as a new geographical record for Sri Lanka. *Colletotrichum siamense* and *C. truncatum* have been identified as new host records on *Eichhornia crassipes*. Isolation of *Phyllosticta capitalensis* on *Nymphaea nouchali* is in confirmative with the findings of Dissanayake et al. (2016) based on multilocus phylogenetic analyses. Furthermore, the study assessed the potential of these endophytic fungi to produce various extracellular enzymes by qualitative assays for amylolytic, cellulolytic, and laccase activities.

## 2 Materials and methods

### 2.1 Sampling, isolation and characterization of endophytic fungi

**Figure 1:**
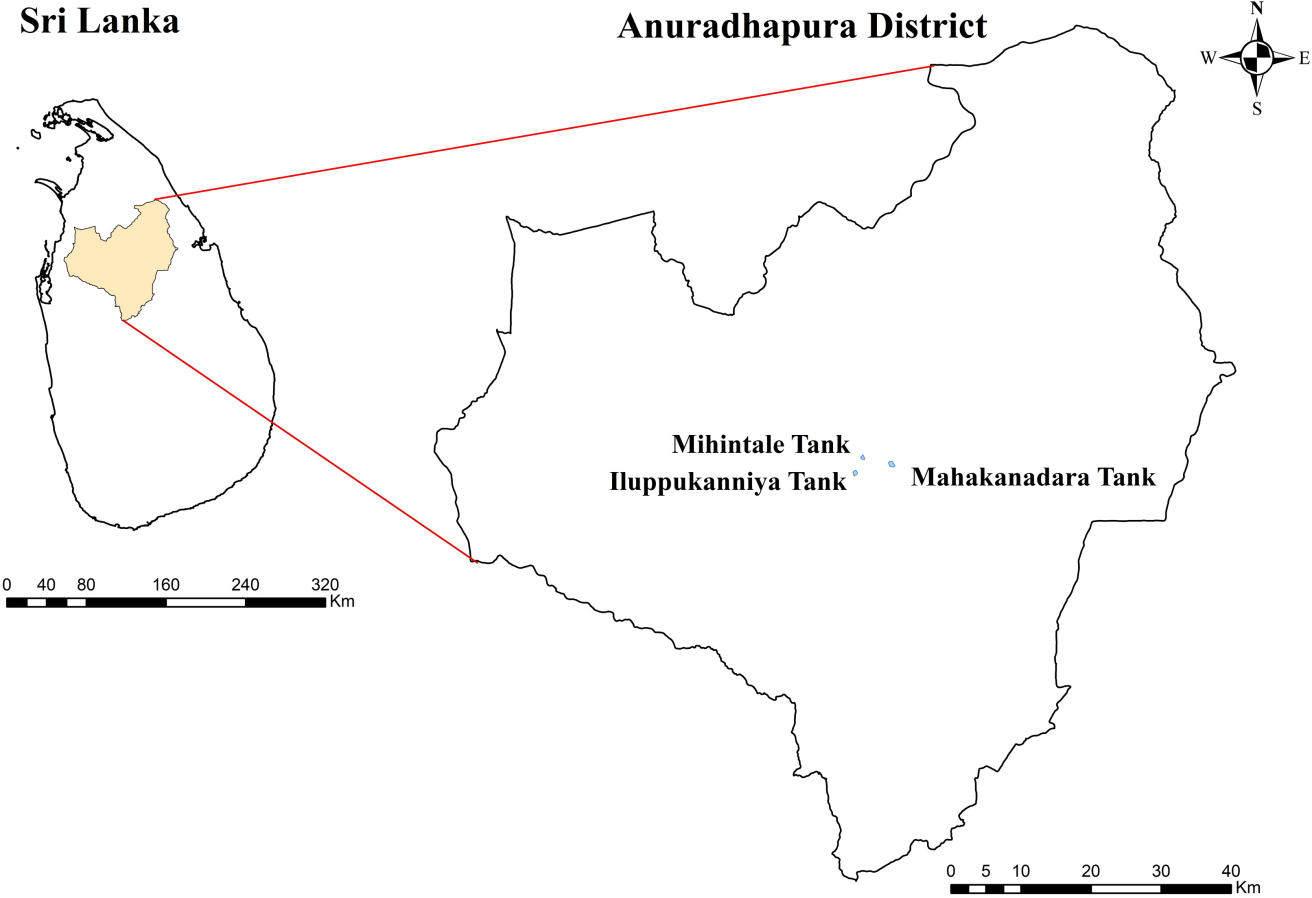
Sampling locations of lentic freshwater habitats in the Mihintale area.

**Figure 2:**
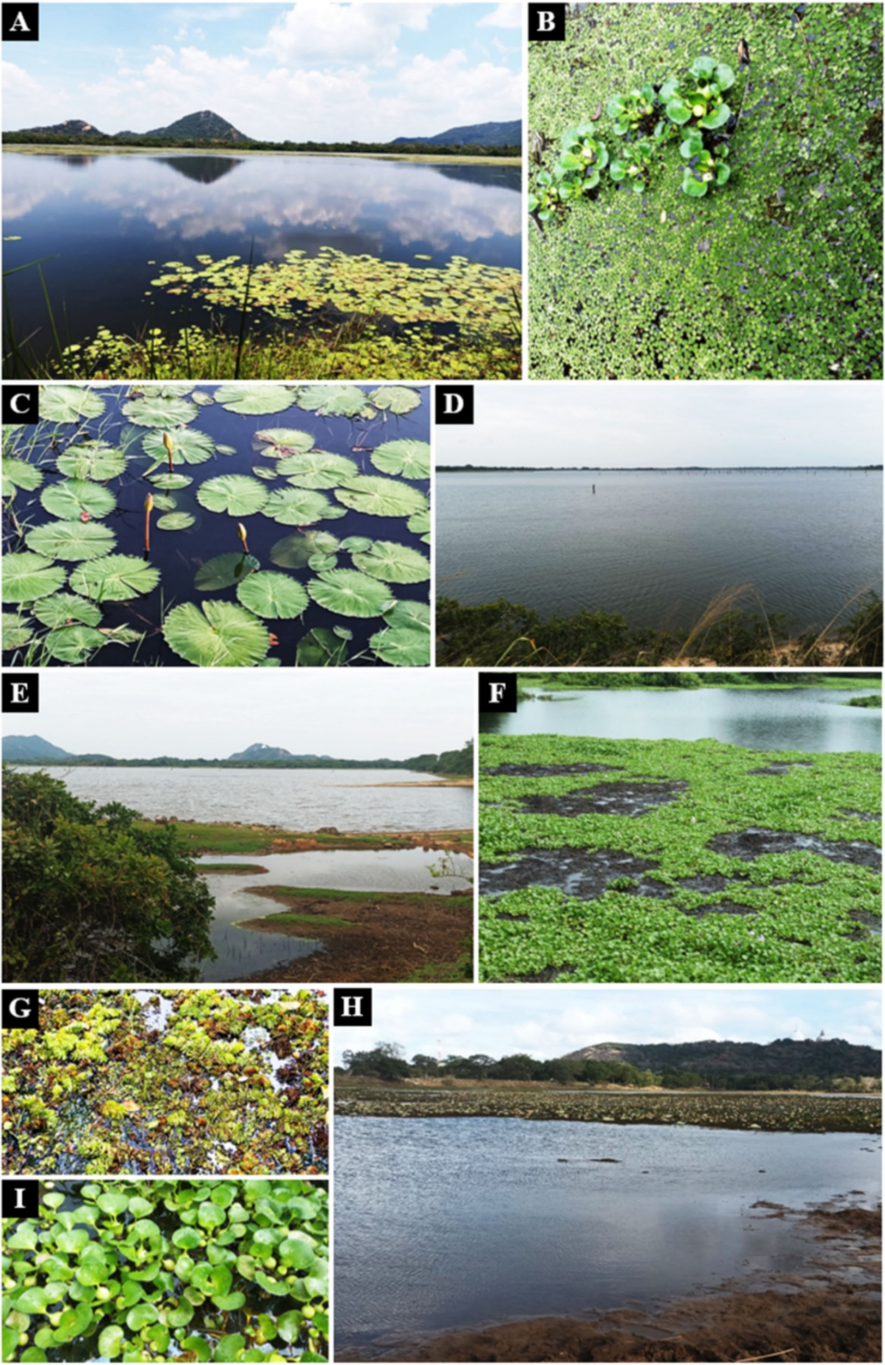
Aquatic plants in lentic freshwater habitats in Mihintale area were used to isolate endophytic fungal species. **(A)** Iluppukanniya tank. **(B)** *Eichhornia crassipes* (Water hyacinth) and S*alvinia minima* (Watermoss) in the Iluppukanniya tank. **(C)** *Nymphaea* sp. in the Iluppukanniya tank. **(D, E)** Mahakanadara tank. **(F)** *Eichhornia crassipes* in the Mahakanadara tank. **(G)** *Salvinia molesta* (Giant Salvinia) in the Mahakanadara tank. **(H)** Mihintale tank. **(I)** *Eichhornia crassipes* in Mihintale tank.

From November to December 2023, healthy aquatic plants were sampled from three lentic freshwater habitats in Mihintale, located in the Anuradhapura district of Sri Lanka including the Iluppukanniya tank (8.36482° N, 80.50764° E, 118 m), Mahakanadara tank (8.38683° N, 80.38683° E, 117 m), and Mihintale tank (8.36267° N, 80.50591° E, 108 m) (**Figure 1**; **Table 1**). Mature plants with undamaged leaves of *Eichhornia crassipes, Nymphaea nouchali, Nymphaea pubescens, Salvinia minima*, and *Salvinia molesta* (**Figure 2**), were carefully uprooted and brought to the laboratory within one hour in ziplock plastic bags containing fresh water. The samples were maintained separately in freshwater until the isolation process began immediately.

**Table 1:** Lentic freshwater habitats and host substrates for the endophytic fungi.

Locations	Host or substrates
Iluppukanniya tank (8.36482° N, 80.50764° E, 118 m)	Leaf of *Eichhornia crassipes* (Water hyacinth) Leaf of *Salvinia minima* (Watermoss) Leaf of *Nymphaea nouchali* (Blue Water-Lily)
Mahakanadara tank (8.38683° N, 80.38683° E, 117 m)	Leaf of *Eichhornia crassipes* (Water hyacinth) Leaf of *Salvinia molesta* (Giant Salvinia)
Mihintale tank (8.36267° N, 80.50591° E, 108 m)	Leaf of *Eichhornia crassipes* (Water hyacinth)

Each plant sample was thoroughly rinsed for 30 seconds under running tap water to remove the debris and adhered mud contaminants. The plants were cut into roots, stems, and leaves and processed in the following sequential surface sterilization steps: an initial immersion in 0.5% sodium hypochlorite for 2 minutes, followed by a rinse in sterile distilled water for 1 minute, immersion in 75% ethanol for 2 minutes, and a final rinse in sterile distilled water for 1 minute. After the final wash, the samples were dried using sterile paper towels under a laminar airflow cabinet (Li et al., 2010; Zheng et al., 2021). However, during surface sterilization of the plant material, the duration of treatment for each plant species depended on its tissue sensitivity. Specifically, being very spongy, the leaves of *Salvinia molesta* were processed with an initial immersion of 1 minute in 0.5% sodium hypochlorite, followed by a rinse in sterile distilled water for one minute, immersion in 70% ethanol one minute, and a final rinse in sterile distilled water for one minute.

Surface-sterilized plant tissues were cut into 5 × 5 mm segments and placed in Potato Dextrose Agar (PDA; 20% potato, 2% dextrose, 2% agar) media supplemented with tetracycline (50 mg/L). The plates were incubated under aseptic conditions at room temperature (28–30°C) and observed for fungal growth every second day for seven days. Once fungal mycelium emerged from the edges of the plant segments, a portion of the growing colony was aseptically cut and transferred to a freshly prepared PDA plate (Li et al., 2010).

The growth of the subcultures was monitored daily, and colony characteristics, including colour, form, elevation, margin, texture, and dimensions, were recorded for two weeks. The colony colour was identified by a colour guide (Colour guide; ArtyClick Colors, 2024). The stock cultures were stored in sterile water and agar slants and preserved at 4°C at Rajarata University Fungal Culture Collection (RUFCC) in Sri Lanka.

The fungal cultures were induced to sporulate using different stress stimulation techniques. These included reducing the normal growth temperature (cold treatment), reducing the quantity of PDA volume for each plate to create starvation media (Mattoo and Nonzom, 2022), exposing the plates to UV light for 15 minutes, and placing a double-sterilized pine needle or toothpick on the fungal colony to induce conidiomatal formation (Su et al., 2012). Post-stressors, the plates were incubated for a week under normal light conditions.

The sporulating cultures were studied using a Nikon SMZ18 stereomicroscope, a Nikon TS2R-FL inverted trinocular microscope and a Nikon ECLIPSE Ci upright microscope. Morphological features were examined and documented (Senanayake et al., 2018).

### 2.2 Molecular identification of endophytic fungi

#### 2.2.1 Genomic DNA extraction

The genomic DNA was extracted from the freshly cultured fungi using trimethylammonium bromide (CTAB) method, following the protocol described by Gontia-Mishra et al. (2014) and Hatamzadeh et al. (2024).

#### 2.2.2 Polymerase chain reaction

The targeted primers used in the polymerase chain reaction (PCR) included; Actin (*ACT*; ACT-512F/ACT-783R), Chitin (*CHS-*1; CHS-354R/CHS-79F), Glyceraldehyde 3-phosphate dehydrogenase (*GAPDH*; GDF1/GDR1/Gpd2-LM), Internal transcribed spacers (ITS; ITS5/ITS4), Large subunit nuclear ribosomal DNA (LSU; LROR/LR5R), RNA polymerase II subunit 2 (*rpb*2; fRPB2-5F2/fRPB2-7cR), Small-subunit ribosomal RNA (SSU; NS1/NS4), Translation elongation factor 1 (*tef*1-α; EF1-728F/EF1-986R), and Beta-tubulin (*tub*2; T1/Bt2b), (**Table 2**), each with specific annealing temperatures. The final volume of the PCR reaction was 25 μl, containing 5 μl of DNA template, 2.5 μl of each forward and reward primer, 12.5 μl of HIMEDIA MBT061-100R 2× PCR TaqMixture (mixture of Taq DNA Polymerase, dNTPs, and optimized buffer) and 2.5 μl of ddH_2_O.

**Table 2:** The gene loci utilized in molecular identification techniques for endophytic fungi.

Fungal genera	Gene loci	Primers	Sequence	References
*Colletotrichum* sp.	ITS	ITS5	5′-GGAAGTAAAAGTCGTAACAAGG-3′	(White et al., 1990)
		ITS4	5′-TCCTCCGCTTATTGATATGC-3′	(White et al., 1990)
	*GAPDH*	GDF1	5′-GCCGTCAACGACCCCTTCATTGA-3′	(Guerber et al., 2003)
		GDR1	5′-GGGTGGAGTCGTACTTGAGCATGT-3′	(Guerber et al., 2003)
	*ACT*	ACT-512F	5′-ATGTGCAAGGCCGGTTTCGC-3′	(Carbone and Kohn, 1999)
		ACT-783R	5′-TACGAGTCCTTCTGGCCCAT-3′	(Carbone and Kohn, 1999)
	*CHS*-1	CHS-354R	5′-TGGAAGAACCATCTGTGAGAGTTG-3′	(Carbone and Kohn, 1999)
		CHS-79F	5′-TGGGGCAAGGATGCTTGGAAGAAG-3′	(Carbone and Kohn, 1999)
	*tub*2	T1	5′-AACATGCGTGAGATTGTAAGT-3′	(O’Donnell and Cigelnik, 1997)
		Bt2b	5′-ACCCTCAGTGTAGTGACCCTTGGC-3′	(Glass and Donaldson, 1995)
*Chaetomella* sp.	ITS	ITS5	5′-GGAAGTAAAAGTCGTAACAAGG-3′	(White et al., 1990)
		ITS4	5′-TCCTCCGCTTATTGATATGC-3′	(White et al., 1990)
	LSU	LROR	5′-ACCCGCTGAACTTAAGC-3′	(Vilgalys and Hester, 1990)
		LR5	5′-TCCTGAGGGAAACTTCG-3′	(Vilgalys and Hester, 1990)
	SSU	NS1	5′-GTAGTCATATGCTTGTCTC-3′	(White et al., 1990)
		NS4	5′-CTTCCGTCAATTCCTTTAAG-3′	(White et al., 1990)
*Ectophoma* sp.	ITS	ITS5	5′-GGAAGTAAAAGTCGTAACAAGG-3′	(White et al., 1990)
		ITS4	5′-TCCTCCGCTTATTGATATGC-3′	(White et al., 1990)
	LSU	LROR	5′-ACCCGCTGAACTTAAGC-3′	(Vilgalys and Hester, 1990)
		LR5	5′-TCCTGAGGGAAACTTCG-3′	(Vilgalys and Hester, 1990)
	*rpb*2	fRPB2-5F2	5′-GGGGWGAYCAGAAGAAGGC-3′	(Sung et al., 2007)
		fRPB2-7cR	5′-CCCATRGCTTGYTTRCCCAT-3′	(Liu et al., 1999)
	*tub*2	T1	5′-AACATGCGTGAGATTGTAAGT-3′	(O’Donnell and Cigelnik, 1997)
		Bt2b	5′-ACCCTCAGTGTAGTGACCCTTGGC-3′	(Glass and Donaldson, 1995)
*Phyllosticta* sp.	ITS	ITS5	5′-GGAAGTAAAAGTCGTAACAAGG-3′	(White et al., 1990)
		ITS4	5′-TCCTCCGCTTATTGATATGC-3′	(White et al., 1990)
	*tef*1-α	EF1-728F	5′-CATCGAGAAGTTCGAGAAGG-3′	(Carbone and Kohn, 1999)
		EF1-986R	5′-TACTTGAAGGAACCCTTACC-3′	(Carbone and Kohn, 1999)
	*ACT*	ACT-512F	5′-ATGTGCAAGGCCGGTTTCGC-3′	(Carbone and Kohn, 1999)
		ACT-783R	5′-TACGAGTCCTTCTGGCCCAT-3′	(Carbone and Kohn, 1999)
	*GAPDH*	GDF1	5′-GCCGTCAACGACCCCTTCATTGA-3	(Guerber et al., 2003)
		Gpd2-LM	5’- CCCACTCGTTGTCGTACCA-3’	(Myllys et al., 2002)
*Neottiosporina* sp.	ITS	ITS5	5′-GGAAGTAAAAGTCGTAACAAGG-3′	(White et al., 1990)
		ITS4	5′-TCCTCCGCTTATTGATATGC-3′	(White et al., 1990)
	LSU	LROR	5′-ACCCGCTGAACTTAAGC-3′	(Vilgalys and Hester, 1990)
		LR5	5′-TCCTGAGGGAAACTTCG-3′	(Vilgalys and Hester, 1990)
	SSU	NS1	5′-GTAGTCATATGCTTGTCTC-3′	(White et al., 1990)
		NS4	5′-CTTCCGTCAATTCCTTTAAG-3′	(White et al., 1990)

The PCR amplification was performed with an initial denaturing step at 95°C for 5 min., followed by 40 amplification cycles consisting of a denaturation step at 95°C for 1 min., an annealing step for 1 min., and a final extension step at 72°C for 10 min. The annealing temperatures were set for the gene loci, with the optimum for each: *ACT*: 58°C, *CHS-*1: 58°C, *GAPDH*: 60°C, *tub*2: 55°C, ITS: 54°C, LSU: 55°C, *rpb*2: 56°C, SSU: 55°C, and *tef*1-α: 54°C. All PCR products were visualized by 1% agarose gel (stained with Diamond TM Nucleic Acid Dye) electrophoresis at 80 V/cm for 30 minutes. The gel was visualized under a UV transilluminator to estimate the fragment size.

#### 2.2.3 DNA sequencing

Amplicons were sequenced using both PCR primers and DNA sequencing results were acquired through Sanger bidirectional sequencing (GeneLabs Medicals Pvt. Ltd., Sri Lanka). The obtained nucleotide sequences were checked for their quality by reviewing the chromatograms using BioEdit version 7.2. After confirming the quality, the sequences were compared with entries in the GenBank database using the Basic Alignment Search Tool (BLAST) (https://blast.ncbi.nlm.nih.gov; accessed on 03 April 2024) to identify significant alignments with similarity percentages.

### 2.3 Phylogenetic analysis

Closely related sequences were downloaded from GenBank based on blast similarity and recent publications. Multiple gene phylogenetic analyses were conducted for endophytic fungi for *Colletotrichum* sp., ITS, *GAPDH*, *ACT*, *CHS-*1, *HIS* 3, and *tub*2 (Damm et al., 2009). *Chaetomella raphigera* was analyzed using ITS, LSU, and SSU (Suwannarach et al., 2018). *Ectophoma salviniae* sp. nov. underwent analysis with ITS, LSU, *rpb*2 and *tub*2 (Hou et al., 2020a)*. Phyllosticta capitalensis* was analyzed by using ITS, *ACT*, *tef*1-α, and *GAPDH* (Glienke et al., 2011; Wang et al., 2012). *Neottiosporina mihintaleensis* sp. nov. was analyzed using ITS, LSU, and SSU (de Gruyter et al., 2009) (see **Table 2** for the primer details). The phylogenetic trees were constructed via Maximum likelihood (ML) and Bayesian analyses. Maximum likelihood (ML) analysis was constructed by the online portal CIPRES Science Gateway v. 3.3 (Miller et al., 2010), using RAxML-HPC v.8 on XSEDE (8.2.12) tool, with the default settings but adapted: with the GAMMA nucleotide substitution model and 1000 rapid bootstrap replicates. Bayesian analysis was generated from MrBayes v. 3.0b4 (Ronquist and Huelsenbeck, 2003), and the model of evolution was estimated with MrModeltest v. 2.2 (Nylander, 2004). The posterior probabilities (PP) (Rannala and Yang, 1996; Zhaxybayeva and Gogarten, 2002) were determined by the following Markov chain Monte Carlo sampling (MCMC) in MrBayes v.3.0b4 (Huelsenbeck and Ronquist, 2001). Six simultaneous Markov chains were run for 1,000,000 generations, with trees sampled every 100^th^ generation. The preburn was set to 0.25 and the run was automatically stopped when the mean standard deviation of the split frequency reached below 0.01 (Maharachchikumbura et al., 2015). The bootstrap values for maximum likelihood (MLBP) and Bayesian posterior probabilities (BYPP) equal to or greater than 50% and 0.95, are given at the respective branches of each phylogenetic trees (See the **Supplementary Tables 1–5**). GTR+I+G model was selected as the best model based on MrModeltest and was used for the Bayesian analysis.

### 2.4 Taxonomic classification

The higher-level taxonomic classification of each freshwater endophytic fungi was based on Wijayawardene et al. (2022a). Index Fungorum identifiers were obtained from Index Fungorum (2024) for the newly introduced taxa following the requirements mentioned in Art. F5.1 of International Code of Nomenclature for Algae, Fungi, and Plant.

### 2.5 Extracellular enzymatic assay of endophytic fungi

The qualitative analysis of amylolytic, cellulolytic, and laccase enzymatic activities of the endophyte isolates conducted using colourimetric changes in the PDA medium. Petri dishes containing PDA supplemented with tetracycline (1600 µg/mL), preventing bacterial contamination (Elshafie et al., 2019). The specific substrates were incorporated into the PDA media for each enzymatic identification, excluding laccase enzymatic activity. Each enzymatic assay included both negative and positive controls. The negative controls consisted of uninoculated fungal PDA plates supplemented with each substrate and treated with the specific chemicals used in each enzymatic assay. The positive controls, involved inoculating fungal PDA plates supplemented with each substrate and treated with the specific chemicals used in each enzymatic assay. All positive and negative controls were incubated at the required incubation temperatures and time periods (see methodology sections 2.5.1, 2.5.2, and 2.5.3).

#### 2.5.1 Qualitative identification of amylase enzymatic activity

Petri dishes containing PDA supplemented with 1% starch were employed for the experiment. The fungal inoculum, comprising small fragments of mycelium (0.5 × 0.5 cm), was carefully placed in the centre of the PDA Petri dishes. Subsequently, the dishes were incubated at 28–30°C for a duration of seven days to facilitate fungal growth and development. Following the incubation period, 1–2 mL of iodine solution was added to each dish. The dishes were then incubated for an additional hour at 28–30°C. Following this incubation, the dishes were thoroughly washed with distilled water to remove any excess iodine solution. The success of the experiment was determined by observing colourimetric changes. A distinct halo appearing around the fungal colony was indicative of a positive result, highlighting the presence of cellulolytic activity (Robledo-Mahón et al., 2020).

#### 2.5.2 Qualitative identification of cellulase enzymatic activity

The 0.5% (w/v) sodium carboxymethyl cellulose (CMC) (Central Drug House Pvt. Ltd., India) was added to the PDA media to evaluate cellulolytic activity. Small pieces of mycelium (0.5 × 0.5 cm) were then positioned on PDA petri dishes. The prepared fungal plates underwent incubation at 28–30°C for a period of three to five days. The qualitative cellulase activity of fungal isolates was assessed based on their ability to proliferate and create cleared zones around colonies on a solid medium. The surface of the media containing the developed fungal colonies was flooded with 0.1% (w/v) Congo red (Himedia Laboratories Pvt. Ltd., Mumbai, India) solution and incubated for 15 minutes at 28–30°C. Afterwards, the dye was removed with sterile distilled water, and the plates underwent an additional 10-minute incubation period at 28–30°C. Subsequently, the plates were further treated by flooding with 1M NaCl (Daejung Chemicals and Metals Co., Ltd., South Korea) for 5 minutes.

#### 2.5.3 Qualitative identification of laccase enzymatic activity

The small pieces of mycelium (0.5 × 0.5 cm) were placed on a PDA medium and incubated for approximately five days at 28–30°C temperature. The colonized Petri dishes were utilized for adding solutions by droplets at the edge of each colony. Laccase activity was determined using a 0.1 M 1-Naphthol (Research Lab Fine Chem. Industries, Mumbai, India). Following the addition of droplets, Petri dishes were incubated for 24 hours at 28–30°C temperature and changes in the colour of the edge of the colony were considered positive results. The blue-purple appearance was displayed for laccase activity at the edge of each fungal colony (Gramss et al., 1998; Robledo-Mahón et al., 2020).

## 3 Results

### 3.1 Phylogenetic analyses

The taxa for each analysis were selected based on blast similarity and related publications and closely related sequences were downloaded from GenBank (See the **Supplementary Tables 1–5**).

#### 3.1.1 Multi-gene analyses for *Ectophoma*

**Figure 3:**
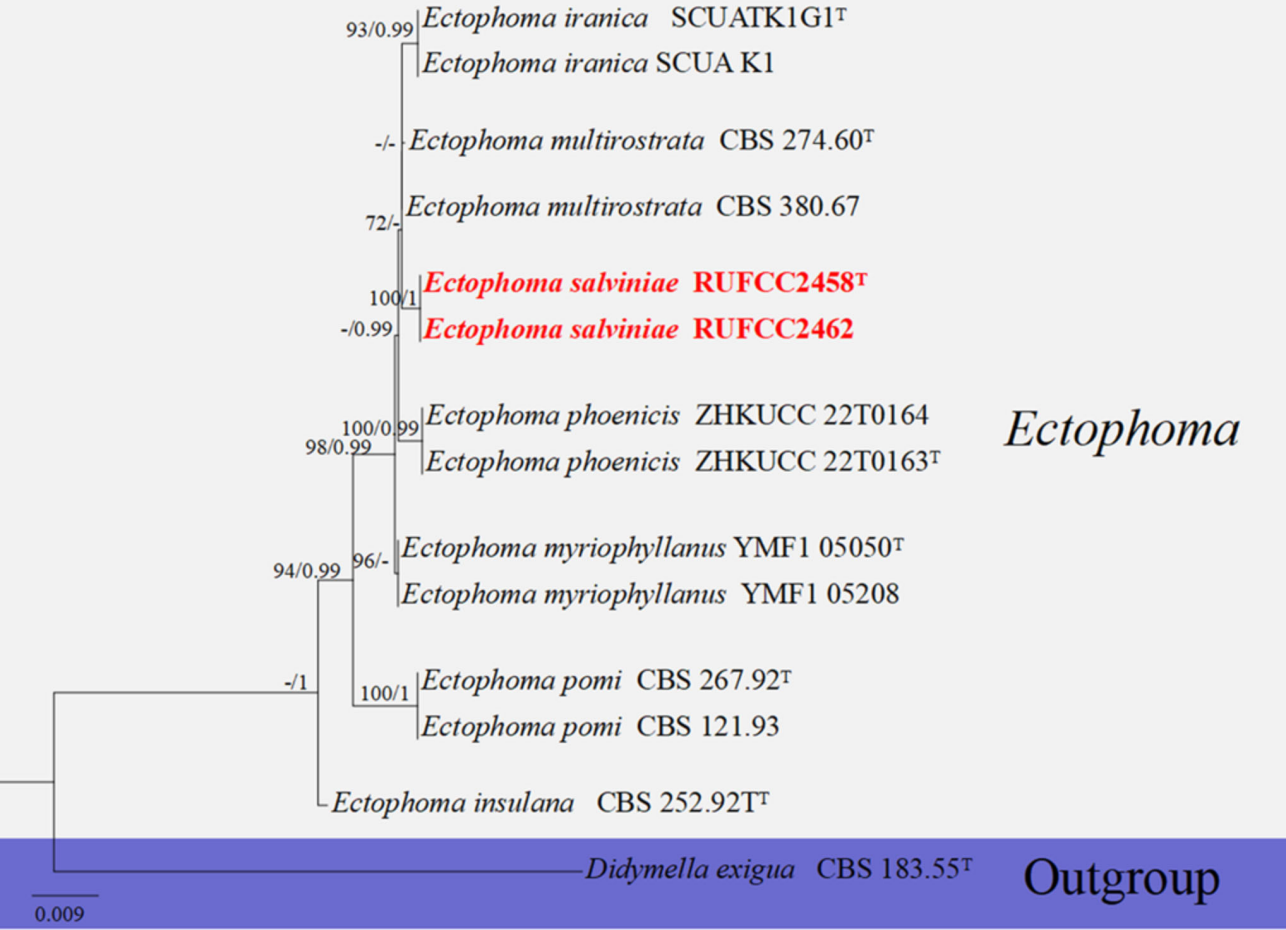
Phylogenetic tree from the best scoring of the RAxML analysis based on combined (ITS, LSU, *rpb*2 and *tub*2) is rooted to *Didymella exigua* (CBS 183.55). Bootstrap values for maximum likelihood (MLBP) and Bayesian posterior probabilities (BYPP) equal to or greater than 50% and 0.95, are given at the respective branches. Hyphen (-) means a value lower than 75% (BS) or 0.95 (PP). Ex-types are marked in “T”. New isolates are labeled in bold and red.

The concatenated dataset of LSU, ITS, *rpb*2, and *tub*2 regions contained 14 isolates, which comprised 2434 characters with gaps. Single gene analysis was carried out to compare the topology of the tree and clade stability. *Didymella exigua* (CBS 183.55) was used as the outgroup taxon. The best-scoring RAxML tree with a final likelihood value of -4415.606556 is presented in **Figure 3**. The matrix had 127 distinct alignment patterns, with 13.83% of undetermined characters or gaps. Estimated base frequencies were as follows: A = 0.236991, C = 0.247923, G = 0.273055, T = 0.242031; substitution rates AC = 2.940086, AG = 5.756609, AT = 1.806664, CG = 1.455718, CT = 18.313095, GT = 1.000000; gamma distribution shape parameter alpha = 0.020000. In the phylogenetic analysis, our new strains (RUFCC2458 and RUFCC2462) form the sister clade to *Ectophoma multirostrata* (CBS 274.60 (ex-type) and CBS 380.67) and *E. iranica* (SCUATK1G1 (ex-type) and SCUAK1) with moderate statistical values (95% ML), 96 PP with BP values more than 95%.

#### 3.1.2 Multi-gene analyses for *Phyllosticta*

**Figure 4:**
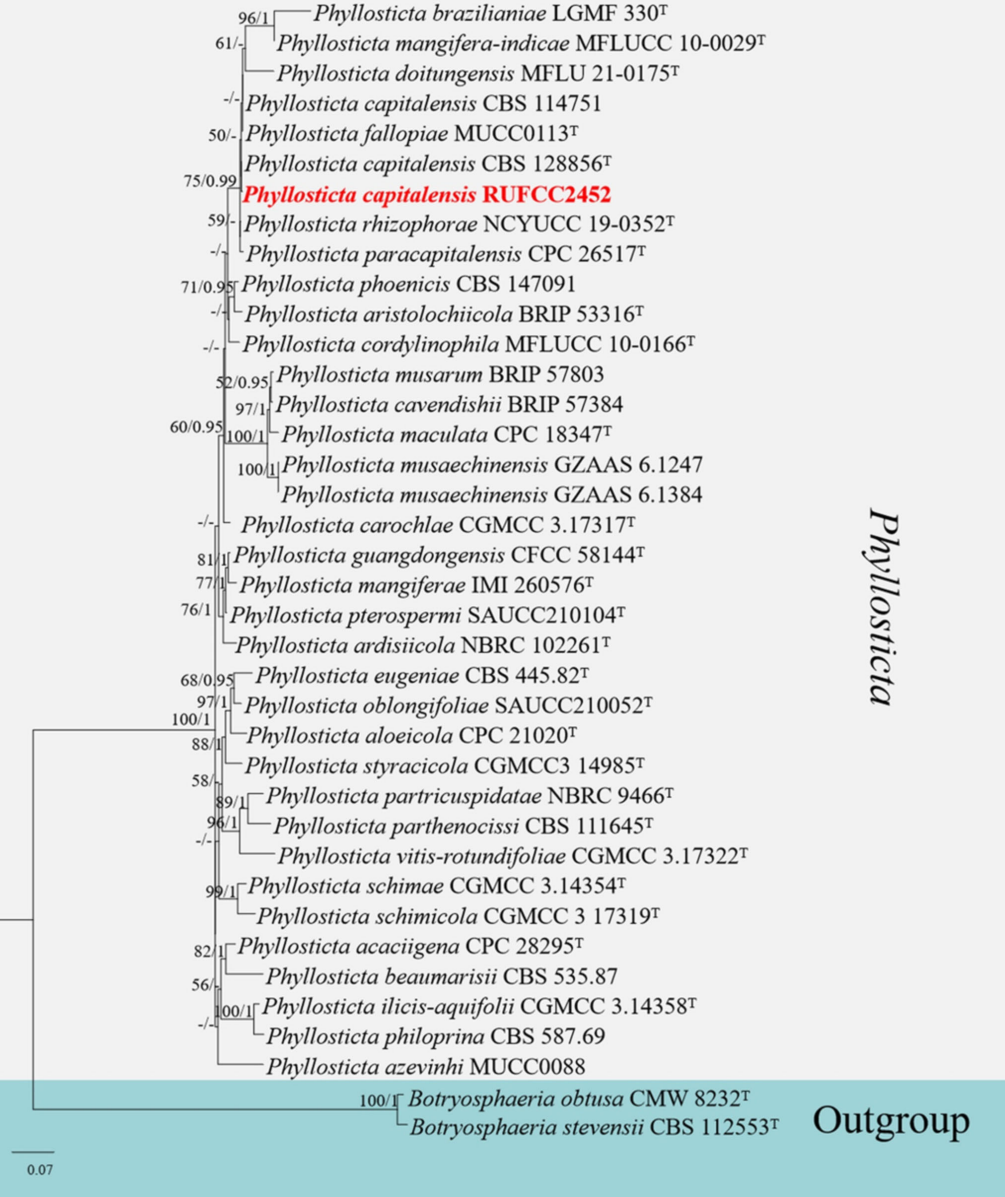
Phylogenetic tree from the best scoring of the RAxML analysis based on combined (ITS, *tef*1-α, *ACT* and *GADPH*) is rooted to *Botryosphaeria obtusa* (CMW 8232) and *B. stevensii* (CBS 112553). Bootstrap values for maximum likelihood (MLBP) and Bayesian posterior probabilities (BYPP) equal to or greater than 75% and 0.95, are given at the respective branches. Hyphen **(-)** means a value lower than 50% (BS) or 0.95 (PP). Ex-types are marked in “T”. New isolates are labelled in bold and red.

The concatenated ITS, *tef*1-α, *ACT* and *GADPH* region dataset contained 38 isolates, comprising 1698 characters with gaps. Single gene analysis was carried out to compare the topology of the tree and clade stability. *Botryosphaeria obtusa* (CMW 8232) and *B. stevensii* (CBS 112553) were used as the outgroup taxa. The best-scoring RAxML tree with a final likelihood value of -9726.371339 is presented in **Figure 4**. The matrix had 727 distinct alignment patterns, with 20.00% of undetermined characters or gaps. Estimated base frequencies were as follows: A = 0.200267, C = 0.311861, G = 0.264840, T = 0.223032; substitution rates AC = 0.893796, AG = 2.729183, AT = 1.229527, CG = 1.044891, CT = 6.112001, GT = 1.000000; gamma distribution shape parameter alpha = 0.344610. The GTR+I+G model was selected as the best model based on MrModeltest and was used for the Bayesian analysis. In the phylogenetic analysis, our new strain (RUFCC2452) clustered with *Phyllosticta capitalensis* (CBS 128856) with high statistical support (75% ML, 0.99 PP).

#### 3.1.3 Multi-gene analyses for *Colletotrichum*

**Figure 5:**
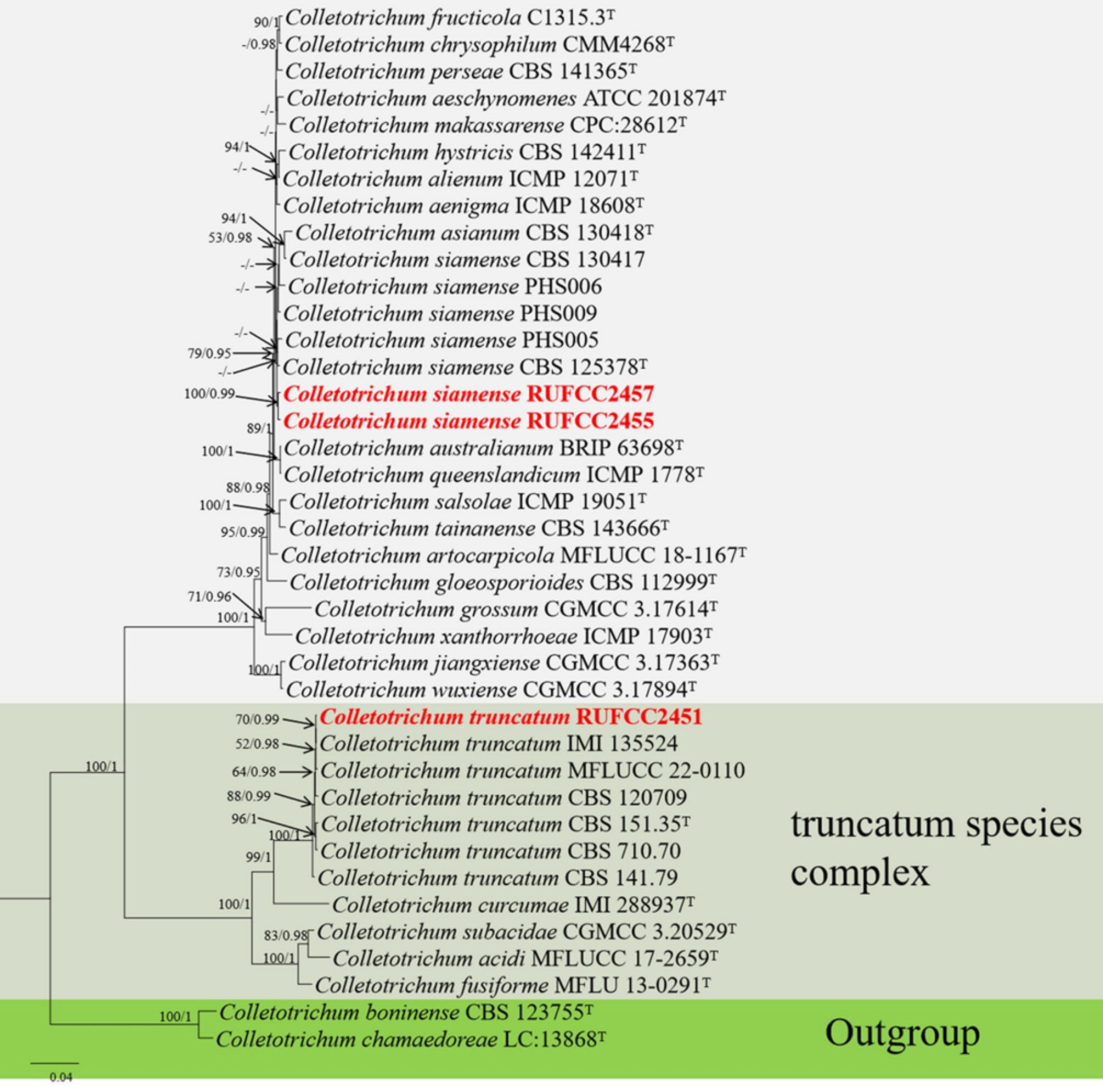
Phylogenetic tree from the best scoring of the RAxML analysis based on combined (ITS, *tub*2, *ACT*, *CHS-1* and *GADP*) is rooted to *Colletotrichum boninense* (CBS 123755) and *C. chamaedoreae* (LC:13868). Bootstrap values for maximum likelihood (MLBP) and Bayesian posterior probabilities (BYPP) equal to or greater than 50% and 0.95, are given at the respective branches. Hyphen (-) means a value lower than 75% (BS) or 0.95 (PP). Ex-types are marked in “T”. New isolates are labelled in bold and red.

The concatenated dataset of ITS, *tub*2, *ACT*, *CHS-*1 and *GADPH* regions contained 39 isolates comprising 1818 characters with gaps. Single gene analysis was carried out to compare the topology of the tree and clade stability. *Colletotrichum boninense* (CBS 123755) and *C. chamaedoreae* (LC:13868) were used as the outgroup taxa. The best-scoring RAxML tree with a final likelihood value of -8361.798032 is presented in **Figure 5**. The matrix had 644 distinct alignment patterns, with 3.72% of undetermined characters or gaps. Estimated base frequencies were as follows: A = 0.232029, C = 0.290209, G = 0.245763, T = 0.231999; substitution rates AC = 1.179354, AG = 3.249038, AT = 1.464001, CG = 0.806083, CT = 5.743800, GT = 1.000000; gamma distribution shape parameter alpha = 0.281325. The GTR+I+G model was selected as the best model based on MrModeltest and was used for the Bayesian analysis. In the phylogenetic analysis, our new strains (RUFCC2457 and RUFCC2455) clustered in the clade that comprises *Colletotrichum siamense* (CBS 125378 (ex-type), *C. australianum* (BRIP 63698), and *C. queenslandicum* (ICMP 1778) with moderate statistical values (79% ML, 0.95 PP). We compared the conidial morphologies of the new collection against the three species and confirmed that our collections belong to *Colletotrichum siamense* (See the taxonomy section). While another new collection (RUFCC2451) clustered in the clade *Colletotrichum truncatum* with high statistical values (100% ML, 1 PP).

#### 3.1.4 Multi-gene analyses for *Chaetomella*

**Figure 6:**
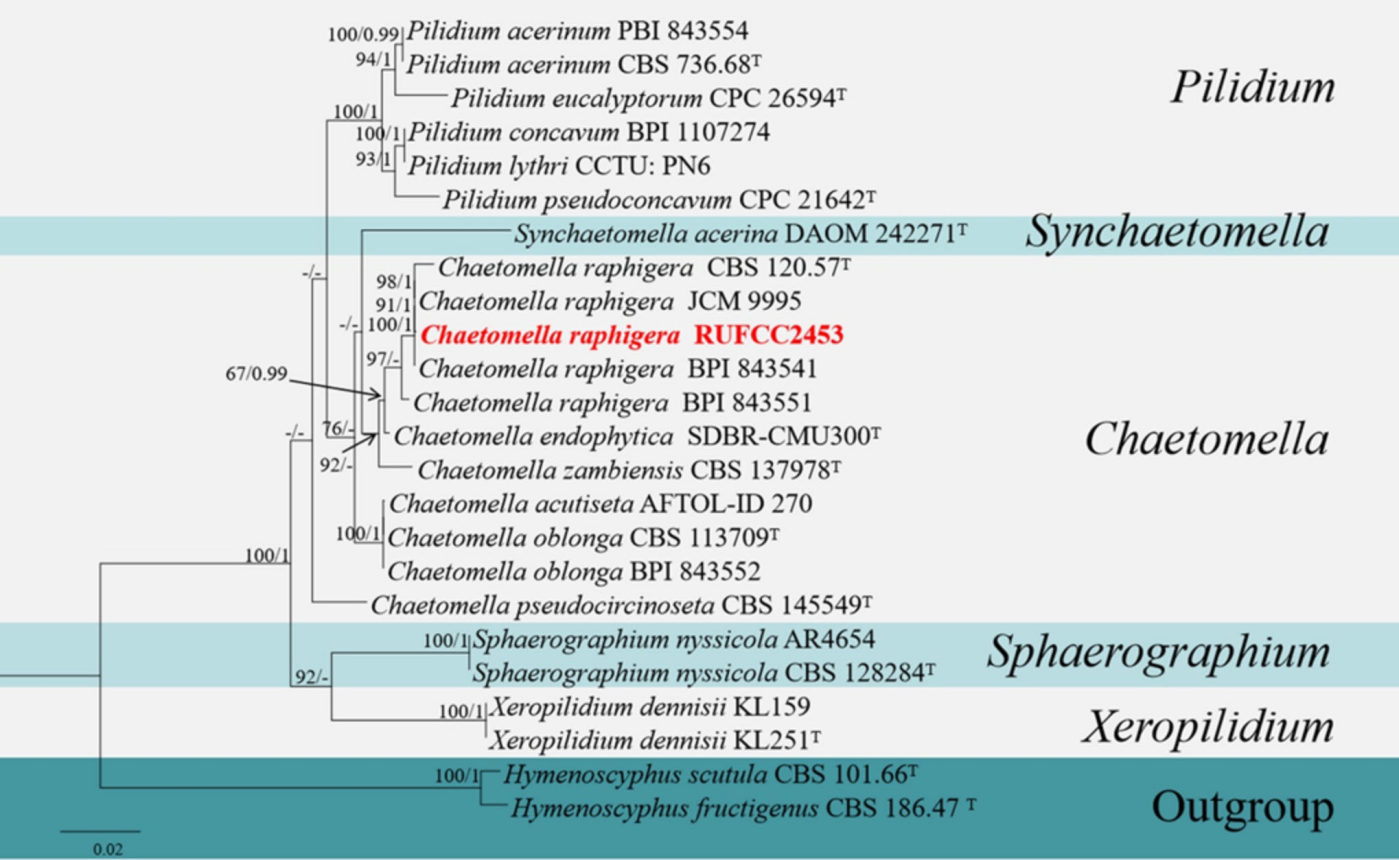
Phylogenetic tree from the best scoring of the RAxML analysis based on combined (LSU, ITS and SSU) is rooted to *Hymenoscyphus scutula* (CBS 101.66) and *H. fructigenus* (CBS 186.47). Bootstrap values for maximum likelihood (MLBP) and Bayesian posterior probabilities (BYPP) equal to or greater than 50% and 0.95, are given at the respective branches. Hyphen (-) means a value lower than 50% (BS) or 0.95 (PP). Ex-types are marked in “T”. New isolates are labelled in bold and red.

The concatenated dataset of LSU, ITS and SSU regions contained 24 isolates, which comprised 2977 characters with gaps. Single gene analysis was carried out to compare the topology of the tree and clade stability. *Hymenoscyphus scutula* (CBS 101.66) and *H. fructigenus* (CBS 186.47) were used as the outgroup taxa. The best-scoring RAxML tree with a final likelihood value of -7269.420511 is presented in **Figure 6**. The matrix had 353 distinct alignment patterns, with 37.79% of undetermined characters or gaps. Estimated base frequencies were as follows: A = 0.263303, C = 0.209012, G = 0.275987, T = 0.251698; substitution rates AC = 1.805316, AG = 2.136673, AT = 0.949986, CG = 0.780819, CT = 4.656160, GT = 1.000000; gamma distribution shape parameter alpha = 0.020000. The GTR+I+G model was selected as the best model based on MrModeltest and was used for the Bayesian analysis. In the phylogenetic analysis, our new strain (RUFCC2453) clustered in the clade *Chaetomella raphigera* with high statistical values (100% ML, 1 PP).

#### 3.1.5 Multi-gene analyses for *Neottiosporina*

**Figure 7:**
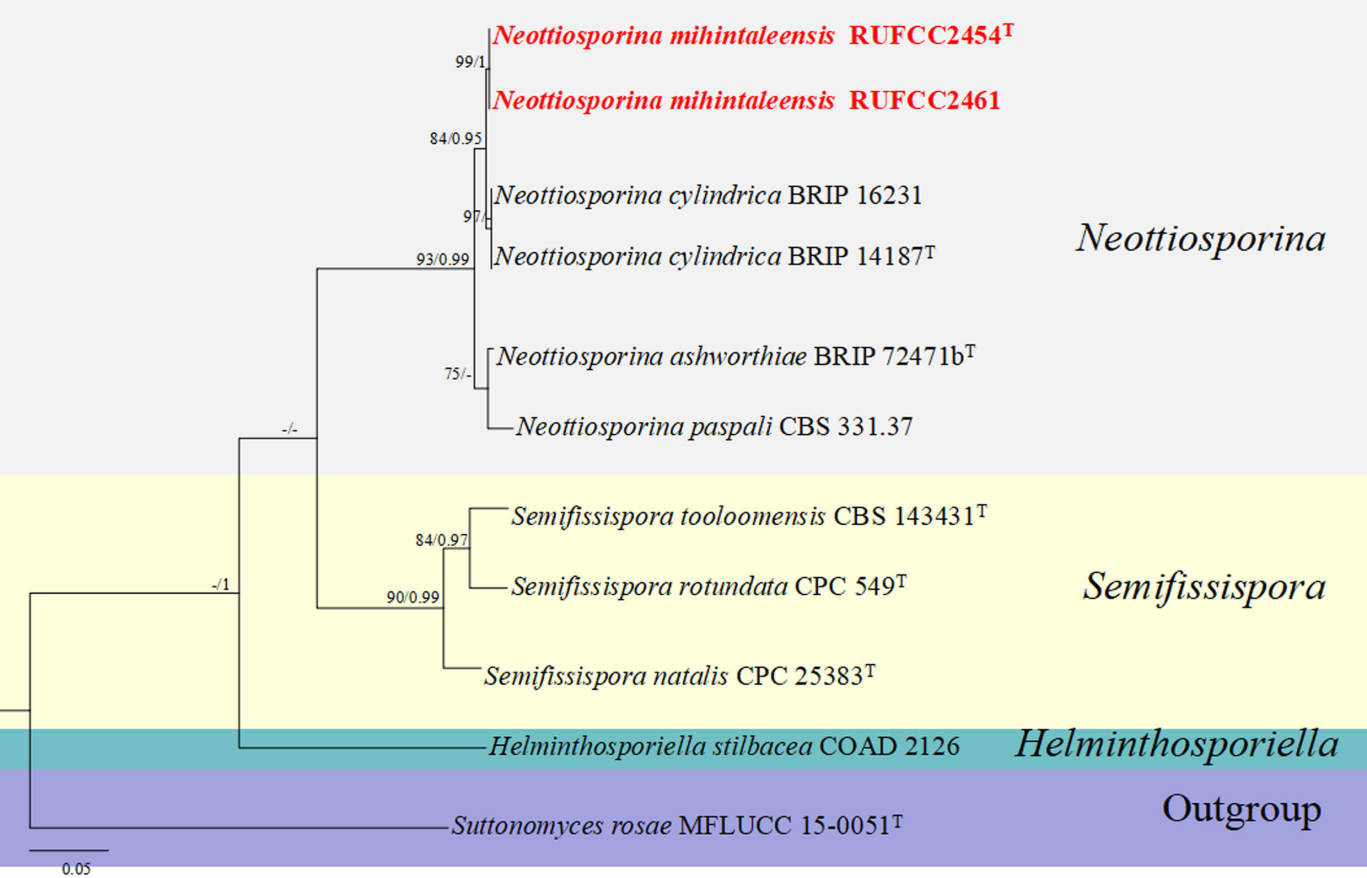
Phylogenetic tree from the best scoring of the RAxML analysis based on combined (ITS) is rooted to *Suttonomyces rosae* (MFLUCC 15-0051). Bootstrap values for maximum likelihood (MLBP) and Bayesian posterior probabilities (BYPP) equal to or greater than 50% and 0.95, are given at the respective branches. Hyphen (-) means a value lower than 50% (BS) or 0.95 (PP). Cultures from holotype and specimens are marked in “T”. New isolates are labelled in bold and red.

The concatenated dataset of ITS regions contained eleven isolates, which comprised 539 characters with gaps. Single gene analysis was carried out to compare the topology of the tree and clade stability. *Suttonomyces rosae* (MFLUCC 15-0051) was used as the outgroup taxon. The best-scoring RAxML tree with a final likelihood value of -1790.869339 is presented in **Figure 7**. The matrix had 138 distinct alignment patterns, with 3.39% of undetermined characters or gaps. Estimated base frequencies were as follows: A = 0.220321, C = 0.266236, G = 0.234288, T = 0.279155; substitution rates AC = 4.232102, AG = 5.947883, AT = 5.637501, CG = 0.698220, CT = 13.019587, GT = 1.000000; gamma distribution shape parameter alpha = 0.164930. The GTR+I+G model was selected as the best model based on MrModeltest and was used for the Bayesian analysis. In the phylogenetic analysis, our new strain (RUFCC2454 (ex-type), and RUFCC2461) form a sister clustered with *Neottiosporina cylindrica* (BRIP 14187 (ex-type) and BRIP (16231) with high statistical values (84% ML, 0.95 PP). Based on the phylogenetic analyses and morphological characters, we confirm our strains differ from *Neottiosporina cylindrica.* Herein, we report our strains as a novel species viz. *Neottiosporina mihintaleensis* sp. nov.

### 3.2 Taxonomy

In this section, we listed all the collected taxa according to the higher-level classification referenced by Wijayawardene et al. (2022a).

*Ascomycota* Caval.-Sm.

*Dothideomycetes* O.E. Erikss. & Winka

*Pleosporales* Luttr. ex M.E. Barr

*Didymellaceae* Gruyter, Aveskamp & Verkley

*Ectophoma* Valenz.-Lopez, Cano, Crous, Guarro and Stchigel, Stud. Mycol. 90: 34 (2017)

Index Fungorum Registration Identifier: 819952

Notes: The genus *Ectophoma* was introduced by Valenzuela-Lopez et al. (2018) with *E. multirostrata* (basionym: *Sphaeronaema multirostratum* P.N. Mathur et al.) as the type species. *Ectophom*a comprises six species in diverse habitats (Valenzuela-Lopez et al., 2018) and a well-defined genus in *Didymellaceae* (Hou et al., 2020a, b). In this study, we introduce *Ectophoma salviniae* sp. nov. from a healthy leaf of *Salvinia minima* (Watermoss).

***Ectophoma salviniae*** Wimalasena, Wijayaw. & Bamunuarachchige sp. nov.

**Figure 8:**
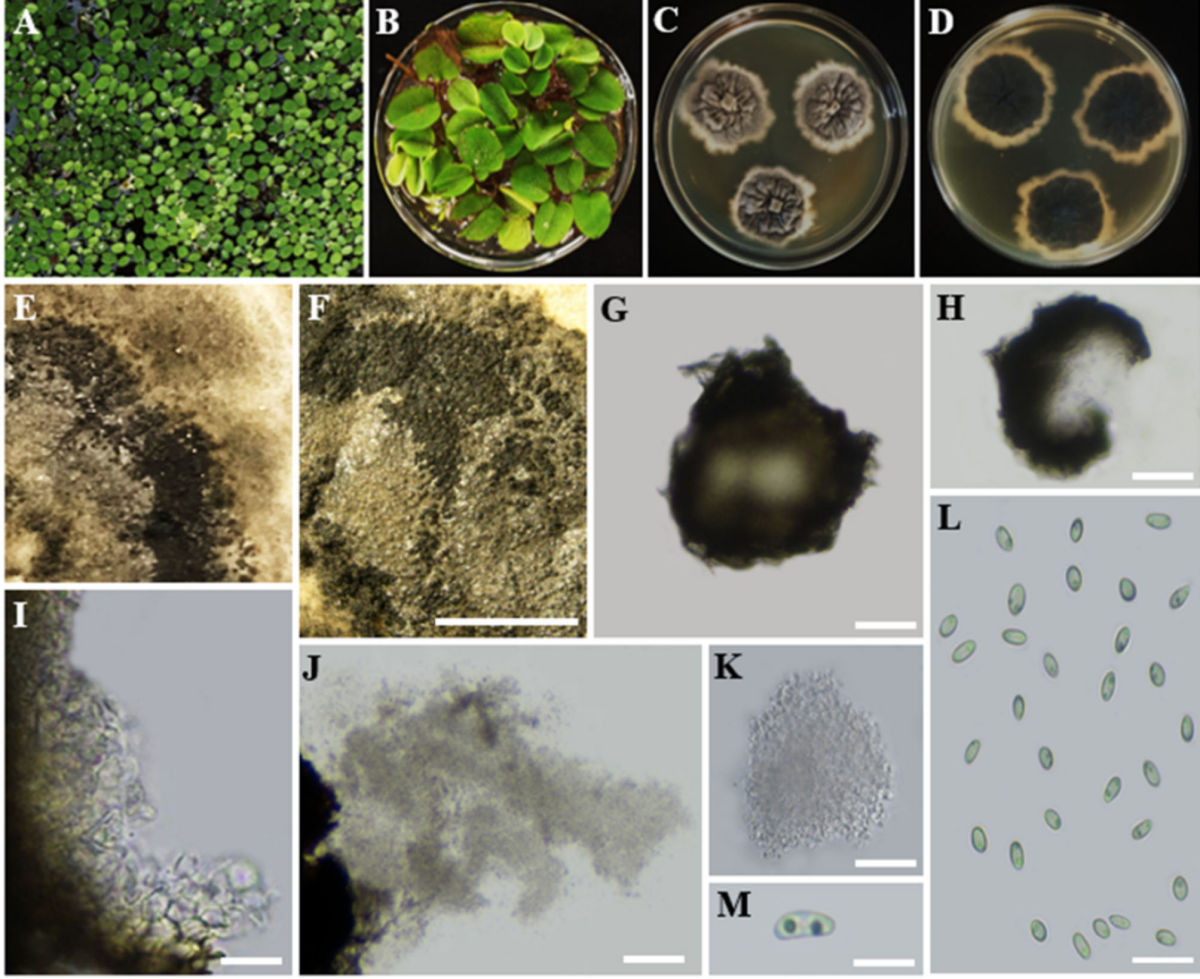
*Ectophoma salviniae* sp. nov. **(A, B**). *Salvinia minima* in the lake. **(A)** and in the lab **(B)** (Watermoss). **(C)** Top view of the PDA culture plate (diameter; 9.5 cm). **(D)** Downside of the PDA culture plate (diameter; 9.5 cm) after five days. **(E)** Sporulated culture after seven days. **(F)** Stereo microscopic view of sporulation in culture. **(G, H)** Squashed pycnidium. **(I)** Inner wall of pycnidium. **(J)** Conidial production. **(K)** Conidiogenous cells and conidia. **(L, M)** Conidia. Scale bars: **(F)** = 1000 µm, **(G–M)** = 100 µm.

Index Fungorum Registration Identifier: IF902503 (**Figure 8**).

Etymology: The name is derived from the host genus *Salvinia*, from which the fungus was isolated.

Holotype: RUSLH/240

Description: Endophytic of healthy leaf of *Salvinia minima.* Sexual morph: Undetermined. Asexual morph on the culture: *Colonies* on PDA slow growing, effuse, with a regular margin, flat, white to grey olivaceous reaching a diam of 1.5–2 cm after 7 days at 28°C. *Mycelium* regular, composed of filamentous, septate, branched, smooth, pale olivaceous hyphae 0.5 µm wide. *Conidiomata* pycnidial, 140–300 µm long × 50–80 µm wide, brown to dark brown, solitary or confluent, abundant, ostiolate, with one or more short necks. *Pycnidial wall*, glabrous, with globose to subglobose or irregular cells of *textura angularis*. *Conidiogenous cells* holoblastic to phialidic, minute. *Conidia* 6.1–9.6 × 3.6–6.7 ( $\bar{\text{x}}$ = 8.0 × 5.0 µm; n = 30) µm, aseptate, hyaline, smooth-walled, oblong to ellipsoidal, end of conidia is acute, guttulate: two guttules are inside the conidial cytoplasm.

Culture characteristics: Colonies on PDA reached a diameter of 1.5–2 cm after 7 days at 28°C, with a regular margin and flat, colourless to weak olivaceous, poorly developed, white to grey olivaceous aerial mycelium. The centre of the colony is olivaceous, followed by a black circle formed by abundant pycnidia. The reverse of the colony was dark black and featured some radially furrowed zones and concentric circles of greyish-black colours.

Material examined: SRI LANKA, North Central Province, Mihintale, Iluppukanniya Tank (8.36482° N, 80.50764° E, 118 m), on healthy leaf of *Salvinia minima* (Watermoss), 28 November 2023, Madhara K. Wimalasena, RUSLH/240 (dried culture as the holotype), RUFCC2462 (ex-type).

Notes: The multi-locus analyses of combined data set of ITS, LSU, *rpb*2, and *tub*2 sequence data revealed that our isolates (RUFCC2458, RUFCC2462) are clustered within the *Ectophoma s. str.*, forming a sister clade to *E. iranica* (SCUATK1G1 (ex-type) and SCUAK1) and *E. multirostrata* (CBS 274.60 (ex-type) and CBS 380.67). The conidiomata of *E. salviniae* show a slight similarity in shape and the dimensions to those of *E. iranica* and *E. multirostrata*. However, *E. salviniae* has larger conidia than in both *E. iranica* and *E. multirostrata* (**Table 3**). Furthermore, its cultural characteristics, including dark greyish to black colonies, are also distinct from *E. iranica* and *E. multirostrata* (**Table 3**). Moreover, phylogenetic analysis further confirms that *E. salviniae* is distinct from *E. iranica* and *E. multirostrata* (**Figure 3**). Herein, the taxon, represented by RUFCC2458 (ex-type) and RUFCC2462 is introduced as a novel species *viz*., *Ectophoma salviniae* sp. nov.

**Table 3:** Diagnostic characters of *Ectophoma iranica, E. multirostrata* and *E. salviniae*.

Morphological and colony characters	Species name and references		
	*E. iranica* (Kularathnage et al., 2023; Larki et al., 2019)	*E. multirostrata* (Ahmadpour et al., 2021; Boerema, 2004)	*E. salviniae* sp. nov. (This study)
Conidiomata	Pycnidia 145.9–382.7 µm long, hyaline to pale brown to brown, with age becoming blackish brown, variable in shape, mostly globose to subglobose but also ovoid, lemon‐shaped	Pycnidia more than 550 µm in diameter, globose to subglobose or irregular. Conidial matrix whitish to cream or buff-coloured	Pycnidia 140–300 µm long × 50–80 µm wide, brown to dark brown, glabrous, globose to subglobose or irregular
Conidiogenous cells	Conidiogenous cells discrete, hyaline, smooth‐walled, globose, phialidic. Dimensions of the conidiogenous cell were not reported	Conidiogenous cell were not reported	Conidiogenous cells were not observed.
Conidia	2.2–5.8 μm long aseptate, oblong to ellipsoidal	Variable in dimensions, mostly 3.8–4.2 × 1.8–2.4 µm, oblong to ellipsoidal, sometimes eguttulate	Conidia 6.1–9.6 × 3.6–6.7 µm ( $\bar{\text{x}}$ = 8.04 × 5.0 µm; n = 30) µm aseptate, hyaline, smooth and thin walled, oblong to ellipsoidal, guttulate
Colony characters	Pale brown to greyish brown colonies	Colourless to weakly olivaceous, white to grey olivaceous to olivaceous buff; reverse olivaceous	Drak greyish to black colour colonies; reverse dark black

*Botryosphaeriales* C.L. Schoch, Crous & Shoemaker

*Phyllostictaceae* Fr.

*Phyllosticta* Pers., Traité champ. Comest. (Paris): 55, 147 (1818)

Index Fungorum Registration Identifier: 9384

= Guignardia Viala & Ravaz, Bull. Soc. mycol. Fr. 8(2): 63 (1892)

Notes: *Phyllosticta* is a well-established genus in *Phyllostictaceae*, *Botryosphaeriales* with over 3000 species epithets in the Index Fungorum 2024 (accession date: 14 of May 2024). This genus currently includes 1,499 recognized species (Sui et al., 2023). Recently, Gong et al. (2024) introduced two novel species (*P. savannaensis* and *P. ovalina*), and Jiang et al. (2024) added three new species (*P. fujianensis, P. saprophytica*, and *P. turpiniae*) to this genus. The members of *Phyllosticta* have been mostly reported as pathogens, saprobes, and endophytes of different hosts worldwide (Wikee et al., 2013; Rodrigues et al., 2019; Sui et al., 2023; Jiang et al., 2024). In this study, we isolated *Phyllosticta capitalensis* as an endophytic taxon from *Nymphaea nouchali.*

*Phyllosticta capitalensis* Henn., Hedwigia 48: 13 (1908) [1909]

**Figure 9:**
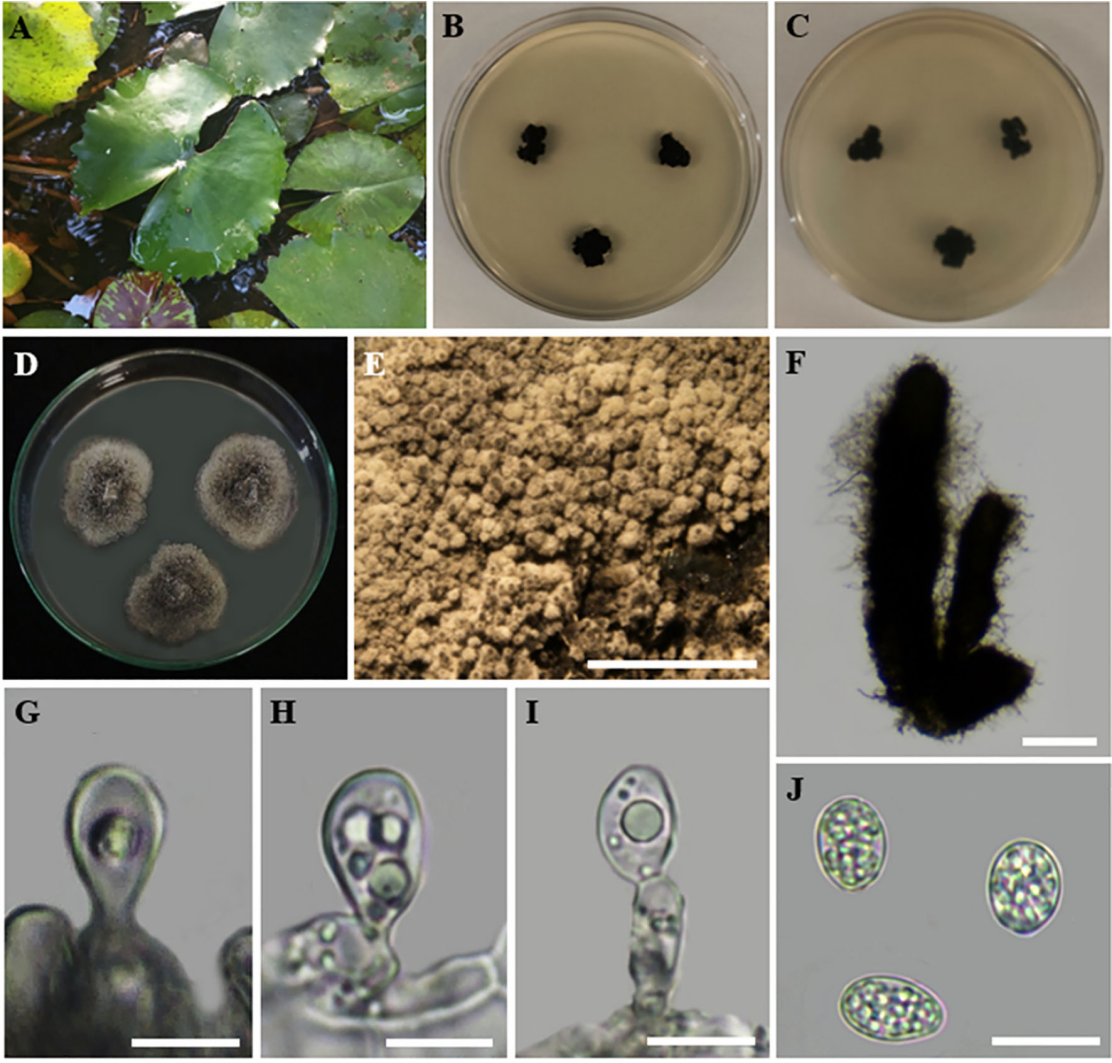
*Phyllosticta capitalensis* **(A)** Host freshwater plant leaf of *Nymphaea nouchali* (Blue Water-Lily). **(B)** Upside of the PDA culture plate (diameter; 9.5 cm). **(C)** Downside of the PDA culture plate (diameter; 9.5 cm) after five days. **(D)** Sporulated culture after seven days. **(E)** Stereo microscopic view of sporulation in culture. **(F)** Conidiomata. **(G-I)** Conidiogenous cells. **(J)** Conidia. Scale bars: **(E)** = 1000 µm, **(F)** = 50 µm **(G-J)** = 100 µm.

Index Fungorum Registration Identifier: 168326 (**Figure 9**).

Description: Endophytic of healthy leaf of *Nymphaea nouchali.* Sexual morph: Undetermined; Asexual morph: *Pycnidia* up to 300 µm diam, 250 µm tall, black, aggregated, erumpent, globose to ampulliform, ostiolate, exuding a colourless, glossy, slimy conidial mass. *Pycnidial wall* consisting of 6–8 layers, up to 40 μm thick, with cells of *textura angularis*. *Ostiole* single, central, 5–15 μm diam. *Conidiophores* subcylindrical to ampulliform, frequently reduced to conidiogenous cells, or branching from a basal supporting cell, coated in mucoid layer, 7–20 × 3–7 μm. *Conidiogenous cells* subcylindrical to ampulliform to doliiform, holoblastic, polyblastic, hyaline, smooth, 7–10 × 3–5 μm; percurrently proliferating 1–2 times near apex. *Conidia* 10–11 × 6–7 μm ( $\bar{\text{x}}$ = 9.5 × 6.5 µm; n = 30), solitary, hyaline, aseptate, thin- and smooth-walled, coarsely guttulate, ellipsoid to obovoid, tapering toward a narrowly truncate base, enclosed in a mucilaginous 2–4 μm thick sheath, bearing a hyaline, mucoid, straight to curved, unbranched, 6–8 × 1–1.5 μm, apical appendage on a bluntly rounded apex.

Culture characteristics: Colonies incubated for 14 days at 28–30°C in darkness on PDA initially appear woolly and white with abundant mycelium. Over the next 2–3 days, they transform from greenish to dark green, with white hyphae visible along the undulating margins, eventually becoming black. Over two weeks of incubation in darkness at 28°C, the mycelium extends to the edge of the Petri dish.

Material examined: SRI LANKA, North Central Province, Mihintale, Iluppukanniya tank (8.36482° N, 80.50764° E, 118 m), on healthy leaf of *Nymphaea nouchali* (Blue Water-Lily), 02 December 2023, Madhara K. Wimalasena, RUFCC2452 (living culture), RUSLH/242 (dried culture as the herbarium specimen).

Notes: *Phyllosticta capitalensis* is often found as an endophyte on a wide range of hosts and exhibits a broad geographic distribution (Silva and Pereira, 2007; Silva et al., 2008; Glienke et al., 2011; Wang et al., 2023). It was reported from 70 plant families and is considered a weak plant pathogen (Wikee et al., 2013). *Phyllosticta capitalensis* has been previously reported in Sri Lanka as a rubber foliar pathogen (Herath et al., 2019) and as an endophytic fungus in the leaves of *Camellia sinensis* (Thambugala et al., 2018). Dissanayake et al. (2016) reported *Phyllosticta capitalensis* on healthy specimens of *Nymphaea nouchali* collected from an unpolluted natural freshwater pond in the Western Province of Sri Lanka, based on a single gene locus (ITS) study. In this study, we reconfirmed the occurrence of *Phyllosticta capitalensis* on healthy leaves of *Nymphaea nouchali* in lentic freshwater habitats in the North-Central Province of Sri Lanka, based on four gene loci (ITS, *tef*1-α*, ACT*, and *GADPH*) study, a polyphasic approach. Previous reports of *Phyllosticta* species in freshwater plants worldwide include *Phyllosticta aquatica* (on *Lemna minor fide* (Spegazzini, 1881), *P. fatiscens* (on *Nymphaea odorata fide* (Anonymous, 1960), and *P. nymphaeacea* (on *Nymphae*a sp. *fide* (Ellis and Everhart, 1900). According to (Farr et al., 2012), *P. capitalensis* has not been reported from *Nymphaea nouchali* so far, and thus, this is the first confirmative report of *P. capitalensis* on *Nymphaea nouchali.* When comparing the recently isolated *P. capitalensis* (SDBR-CMU497 and SDBR-CMU498) (Chaiwong et al., 2024) isolates with *P. capitalensis* RUFCC2452, their morphological features, such as pycnidia, conidiophores, and conidiogenous cells, are similar. However, the asexual conidia of *P. capitalensis* RUFCC2452 are slightly larger than those of the SDBR-CMU497 and SDBR-CMU498 (5.2 to 9.4 × 3.6 to 7.5 µm (n = 50) isolates.

*Leotiomycetes* O.E. Erikss. & Winka

*Chaetomellales* Crous & Denman

*Chaetomellaceae* Baral, P.R. Johnst. & Rossman

*Chaetomella* Fuckel, Jb. nassau. Ver. Naturk. 23–24: 401 (1870) [1869–70]

Index Fungorum Registration Identifier: 7575

Notes: Fuckel (1869), established the genus *Chaetomella*, including two species; *C. oblonga*, characterized by hyaline spores, and *C. atra*, characterized by olivaceous spores. Among these, the type species was *C. oblonga* (Rossman et al., 2004). The members of the genus were reported as plant pathogens (Gajbhiye et al., 2016; Pärtel et al., 2017; Nguyen et al., 2018; Suwannarach et al., 2018; Cao et al., 2021), saprophytes and as endophyte (Suwannarach et al., 2018). As of June 2024, the Index Fungorum lists 61 records for the genus *Chaetomella* (https://www.indexfungorum.org/names/Names.asp). In this study, we report *C. raphigera* from the healthy leaf of *Eichhornia crassipes* as an endophytic species.

*Chaetomella raphigera* Swift, Mycologia 22(4): 165 (1930)

= *Volutellospora raphigera* (Swift) Thirum. & P.N. Mathur, Sydowia 18 (1–6):38 (1965)

= *Chaetomella terricola* P.Rama Rao, Mycopathologia et Mycologia Applicata 19 (3):255 (1963)

**Figure 10:**
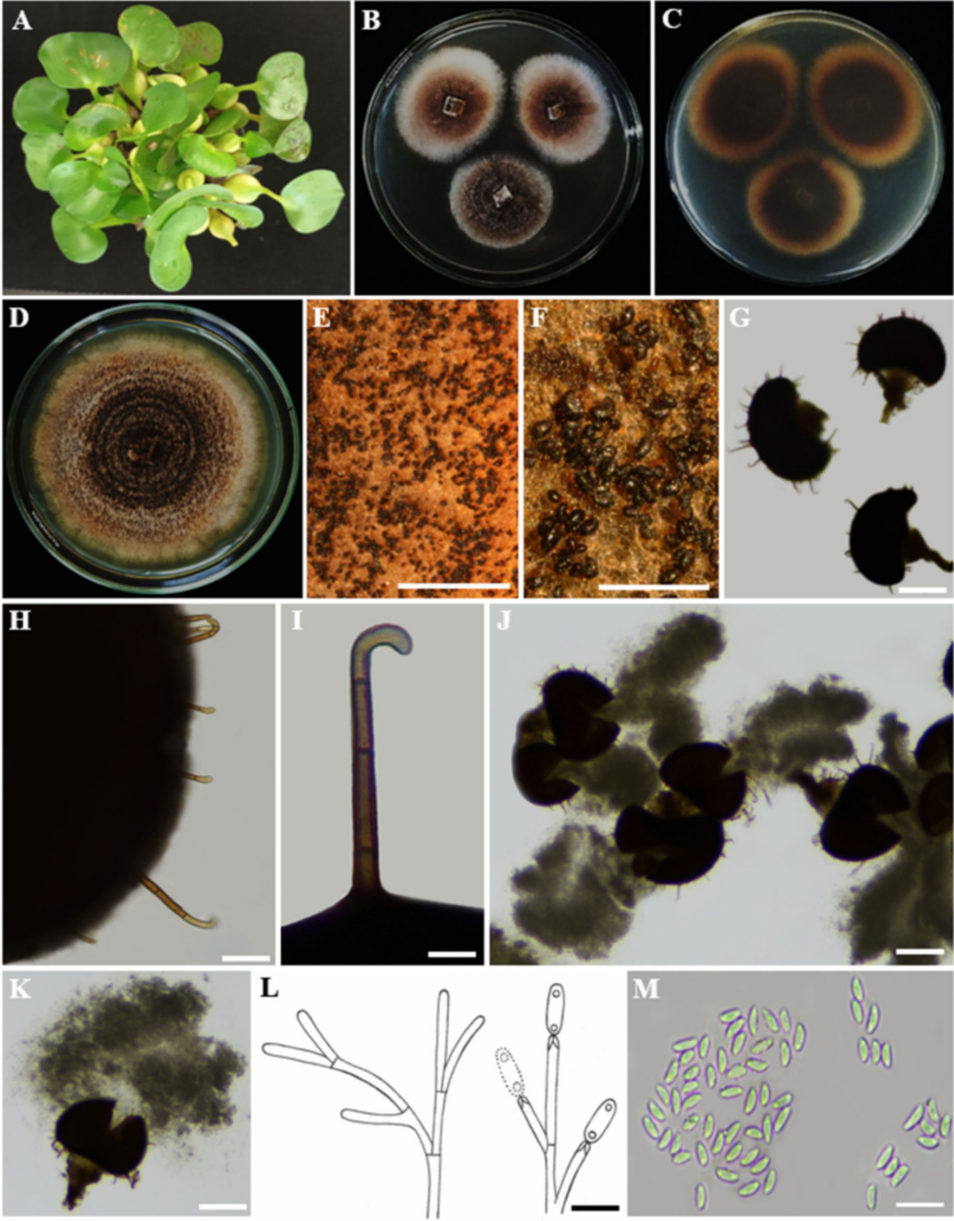
*Chaetomella raphigera* **(A)** Host freshwater plant leaf of *Eichhornia crassipes* (Water hyacinth). **(B)** Upside of the PDA culture plate (diameter; 9.5 cm). **(C)** Downside of the PDA culture plate (diameter; 9.5 cm) after five days. **(D)** Sporulated culture after seven days. **(E, F)** Stereo microscopic view of sporulation in culture. **(G)** Pycnidia. **(H, I)** Types of setae on pycnidium. **(J, K)** Pycnidia release the conidia. **(L)** Conidiogenous cells. **(M)** Conidia. Scale bars: **(E, F)** = 1000 µm **(G–M)** = 100 µm.

Index Fungorum Registration Identifier: 163400 (**Figure 10**).

Description: Endophytic of healthy leaf of *Eichhornia crassipes.* Sexual morph: Undetermined. Asexual morph: *Conidiomata* pycnidial, 200–350×100–250 μm, solitary, short-stipitate, globose to ovate, ostiolate, widely opening lengthwise, dark brown to black, thick-walled, setiferous. Basal stipe short, composed of hyaline, pseudoparenchymatous cells. *Setae* 50–100×2.5–5 μm, brown, smooth, thick-walled, septate, unbranched, with hooked apices. *Conidiophores* hyaline, short, branched, filiform, septate, and smooth. *Conidiogenous cells* enteroblastic, phialidic, determinate, integrated, filiform, hyaline, and smooth. *Conidia* 3.5–6 × 1.–2.3 µm ( $\bar{\text{x}}$ = 4.8 × 1.6 µm; n = 30), hyaline, aseptate, cymbiform to allantoid, on maturity released by splitting the pycnidial wall along the thin-walled cells of the raphe, in mass becoming amber-coloured on aging.

Culture characteristics: Colonies on incubation for 14 days at 28–30°C in darkness on PDA media, attaining 5.5 cm diam., cinnamon brown with yellowish-white margins and with septate, branched mycelium. The reverse of the colony is dark brown in the center with yellowish-white edges. Sporulation is visible in a circular pattern on the surface of the colony.

Material examined: SRI LANKA, North Central Province, Mihintale, Iluppukanniya tank (8.36482° N, 80.50764° E, 118 m), on healthy leaf of *Eichhornia crassipes* (Water hyacinth), 5 December 2023, Madhara K. Wimalasena, RUFCC2453 (living culture), RUSLH/243 (dried culture as the herbarium specimen).

Notes: *Chaetomella raphigera* has been reported as a plant pathogen from India (Gajbhiye et al., 2016). However, this is the first report of *C. raphigera* as a new geographical record in Sri Lanka, found in the freshwater plant *Eichhornia crassipes*. Besides, *Chaetomella* species have not been reported in Sri Lanka thus this is the first genus report from the country. Morphological similarities of *C. raphigera* (RUFCC2453) with previous studies are mentioned in **Table 4**.

**Table 4:** Morphological similarities of *Chaetomella raphigera* (RUFCC2453) with previous studies.

*C. raphigera* strains	Morphological and colony characters			
	Conidiomata	Conidiogenous cells	Conidia	Colony characters
*C. raphigera* (Rossman et al., 2004)	Pycnidia 150–450 × 100–200 µm on natural substratum, 200–320 × 140–200 µm in culture, elongated, reniform, pale to dark reddish brown, with a short stalk of hyaline textura angularis	Conidiogenous cells enteroblastic, collar and channel minute	Conidia non-septate, hyaline, ellipsoid with broadly rounded ends, straight or slightly curved, smooth, guttulate, 5.2–7.5 × 2.0–3.0 µm ( $\bar{\text{x}}$ = 6.41 × 2.47 µm, n = 87)	Colonies 4.3–5.0 cm diam., no aerial mycelium, submerged mycelium cinnamon to dark brick, sporulating profusely
*C. raphigera* (TAC-15/MUBL No. 665), (Gangadevi and Muthumary, 2009)	Conidiomata are pycnidial, separate, globose but opening widely, very shortly stipitate, dark brown to black, thick-walled, 200–350 × 100–250 μm	Conidiogenous cells enteroblastic, phialidic, determinate, integrated, filiform, hyaline, smooth	Conidia hyaline, aseptate, cymbiform to allantoid, 3.75–6.25 × 1.25–2.5 µm	Colonies are brown, septate, branched mycelium
*C. raphigera* (BF79/JX863671 and BF99/KF308287), (Gajbhiye et al., 2016)	Pycnidia were dark reddish brown, oval, approximately 200 µm × 300 µm	Conidiogenous cells not reported	Conidia were produced apically on conidiophores, aseptate, ellipsoidal with rounded ends, smooth, straight or curved, 10–12 µm × 2–3 µm	Colony characters not reported
*C. raphigera* (CNUFC-GHD05-1), (Nguyen et al., 2018)	Elongated, reniform, pale to dark reddish brown, and measured 72.5–148.5 µm × 46.5–88.5 µm	Conidiogenous cells not reported	Ellipsoid, and measured 4.8–7.2 µm × 1.8–2.6 µm	Slowly-growing, white at first, becoming cinnamon brown in age
*C. raphigera* (Cao et al., 2021)	Pycnidia were pale to dark brown, globose or oblate (245.98–491.33 µm × 123.14–274.11 μm), covered with setae	Conidiogenous cells not reported	Conidia were hyaline, oval or boat-shaped (5.19–6.52 µm × 1.87– 2.66 μm)	Colonies were pale brown with rare aerial mycelium and abundant pycnidia production.
*C. raphigera* RUFCC2453 (This study, 2024)	Conidiomata pycnidial, 200–350 µm ×100–250 μm, solitary, short-stipitate, globose to ovate, ostiolate, widely opening lengthwise, dark brown to black, thick-walled, setiferous	Conidiogenous cells enteroblastic, phialidic, determinate, integrated, filiform, hyaline, and smooth	Conidia 3.5–6 µm × 1.–2.3 µm ( $\bar{\text{x}}$ = 4.8 × 1.6 µm; n = 30), hyaline, aseptate, cymbiform to allantoid, on maturity released by splitting the pycnidial wall	Colonies 5.5 cm diam., cinnamon brown with yellowish-white margins and with septate, branched mycelium. The reverse of the colony is dark brown in the center with yellowish-white edges. Sporulation is visible in a circular pattern on the surface of the colony

*Sordariomycetes* O.E. Erikss. & Winka

*Glomerellales* Chadef. ex Réblová, W. Gams & Seifert

*Glomerellaceae* Locq. ex Seifert & W. Gams

*Colletotrichum* Corda

Index Fungorum Registration Identifier: 7737

Note: *Colletotrichum* represents a diverse and complex genus, with currently 344 recognized species grouped into 20 species complexes (Talhinhas and Baroncelli, 2021). These species often lead to considerable economic losses, mostly infecting economically important crops (Peng et al., 2023; Peralta-Ruiz et al., 2023; Zhang et al., 2023). The members of *Colletotrichum* exhibit different lifestyles that are found in varied environments and host species (Jayawardena et al., 2016a, b; Samarakoon et al., 2018; Talhinhas and Baroncelli, 2023). These include necrotrophic (Vargas et al., 2012; De Silva et al., 2017; Talhinhas and Baroncelli, 2021; Páez Redondo et al., 2022), biotrophic and hemibiotrophic (De Silva et al., 2017; Páez Redondo et al., 2022; Jia et al., 2023), quiescent (De Silva et al., 2017; Fu et al., 2022), and endophytic (De Silva et al., 2017; Páez Redondo et al., 2022; Lin et al., 2023; Liu et al., 2023; Zhang et al., 2023; Barreto Ramos et al., 2024) lifestyles. Among these lifestyles, endophytic *Colletotrichum* spp. have been documented in marine environments such as mangroves (Grano-Maldonado et al., 2021; Norphanphoun and Hyde, 2023; Aumentado et al., 2024) and freshwater habitats (Zheng et al., 2022). In this study, we isolated *C. siamense* and *C. truncatum* as two endophytic taxa of *Eichhornia crassipes* in freshwater environments.

*Colletotrichum siamense* Prihast., L. Cai & K.D. Hyde, *Fungal Diversity* 39: 98 (2009)

**Figure 11:**
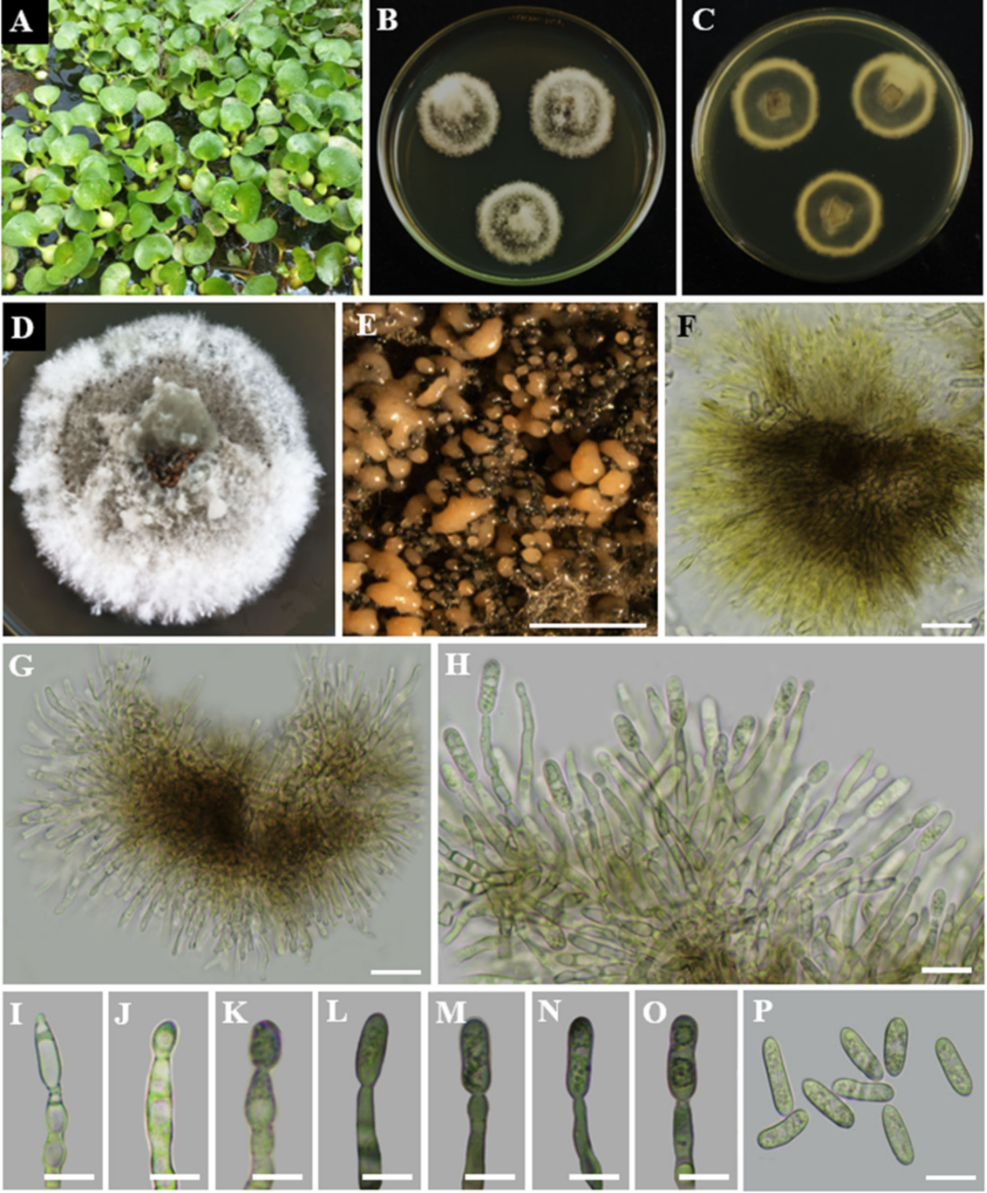
*Colletotrichum siamense* **(A)** Host freshwater plant leaf of *Eichhornia crassipes* (Water hyacinth). **(B)** Upside of the PDA culture plate (diameter; 9.5 cm). **(C)** Downside of the PDA culture plate (diameter; 9.5 cm) after seven days. **(D)** Sporulated colony. **(E)** Acervuli with orange conidial ooze. **(F, G)** Conidiomata and conidiogenous cells. **(H)** Conidiogenous cells. **(I–P)**, Maturity levels of conidiogenous cells. **(P)** Conidia. Scale bars: **(E)** = 500 µm, **(F–P)** = 100 µm.

Index Fungorum Registration Identifier: 515410 (**Figure 11**).

Description: Endophytic of healthy leaf of *Eichhornia crassipes.* Sexual morph: Undetermined. Asexual morph: *Conidiomata* acervular, pulvinate, with erect conidiophores formed on a cushion of roundish and medium brown cells. *Setae* not observed. *Conidiophores* maconematous, hyaline, septate, branched. *Conidiogenous* cells hyaline, cylindrical to ampulliform, phialidic, smooth, guttulate, 7–15.5 μm long, 1–2.5 μm wide at apex. *Conidia* 9.89–15.95 × 3.73–5.67 µm ( $\bar{\text{x}}$ = 13.75 × 4.75 µm; n = 30), hyaline, aseptate, smooth-walled, cylindrical, bluntly rounded at both ends, guttulate.

Culture characteristics: Colonies on incubating for ten days at 28–30°C in dark on PDA media, reaching a diameter of 1–1.5 cm *Eichhornia crassipes*. The aerial mycelium is white, cottony, and sparse. The colony surface features numerous, small acervuli with orange conidial ooze, and the reverse side remain pale yellowish.

Material examined: SRI LANKA, North Central Province, Mihintale tank (8.36267° N, 80.50591° E, 108 m), Mihintale, on healthy leaf of *Eichhornia crassipes* (Water hyacinth), 30 November 2023, Madhara K. Wimalasena, RUFCC2455 and RUFCC2457 (living cultures), RUSLH/244 (dried culture as the herbarium specimen).

Notes: *Colletotrichum siamense* and *C. truncatum* are important plant pathogens causing a wide range of diseases worldwide (Talhinhas and Baroncelli, 2023). Several studies reported *C. siamense* and *C. truncatum* from different hosts and habitats in Sri Lanka and these include *Allium cepa* (Herath et al., 2021), *Hevea brasiliensis* (Herath et al., 2019), *Musa* sp. (Kurera et al., 2023), and *Persea americana* (Dissanayake et al., 2021) as hosts for *Colletotrichum siamense*, while *Begonia* sp. (Wickramasinghe et al., 2019), *Capsicum annuum* (Welideniya et al., 2019), and *Hevea brasiliensis* (Herath et al., 2019) have been identified as hosts for *Colletotrichum truncatum*. De Silva et al. (2019) reported isolates of *Colletotrichum siamense* from different countries showed noticeable differences in growth rates and culture morphology. However, conidial measurements from isolates in distinct subclades of the phylogenetic tree were consistent, and the morphological traits within each subclade were highly uniform within each country (De Silva et al., 2019) (see **Table 5** for the morphological comparison).

**Table 5:** *Colletotrichum siamense* strains reported in different geographical locations and their conidial measurements (CPC-Culture collection of P.W. Crous, housed at Westerdijk Fungal Biodiversity Institute, RUFCC-Rajarata University Fungal Culture Collection, UOM-University of Melbourne culture collection, Victoria, Australia).

*Colletotrichum siamense* strains reported in different geographical locations	Host and distribution	Conidial measurements
*C. siamense* (UOM 1116), (De Silva et al., 2019)	Fruit lesion of *Capsicum* sp., Kandy, Sri Lanka	10.5–16.5 × 3.5–5.5 µm
*C. siamense* (CPC 30233), (De Silva et al., 2019)	Fruit lesion of *Capsicum annuum*, Gowa, Indonesia	12.5–17 × 2.5–5.5 μm
*C. siamense* (UOM 1132), (De Silva et al., 2019)	Fruit lesion of *Capsicum* sp., Ratchaburi, Thailand	9.5–14.5 ×3.5–5 μm
*C. siamense* (UOM 1126/F4-1C), (De Silva et al., 2019)	Fruit lesion of *Capsicum* sp., Kanchana Buri, Thailand	12–15 × 5–7 μm
*C. siamense* (RUFCC2457), This study	On healthy leaf of *Eichhornia crassipes*, Mihintale, Sri Lanka	9.89–15.95 × 3.73–5.67 µm

Huang et al. (2021) reported *C. fructicola* (which causes irregular necrotic lesions on leaves, stems, and crown and petiole rot symptoms) from *Eichhornia crassipes* in China. However, as far as we know, there are no hitherto reports of *C. siamense* or *C*. *truncatum* on *Eichhornia crassipes* in Sri Lanka or elsewhere (2024; accession date: 06 June 2024, https://fungi.ars.usda.gov/). Hence, this is the first host report of *C. siamense* and *C. truncatum* from *Eichhornia crassipes* from Sri Lanka.

*Colletotrichum truncatum* (Schwein.) Andrus & W.D. Moore, Phytopathology 25: 121 (1935)

**Figure 12:**
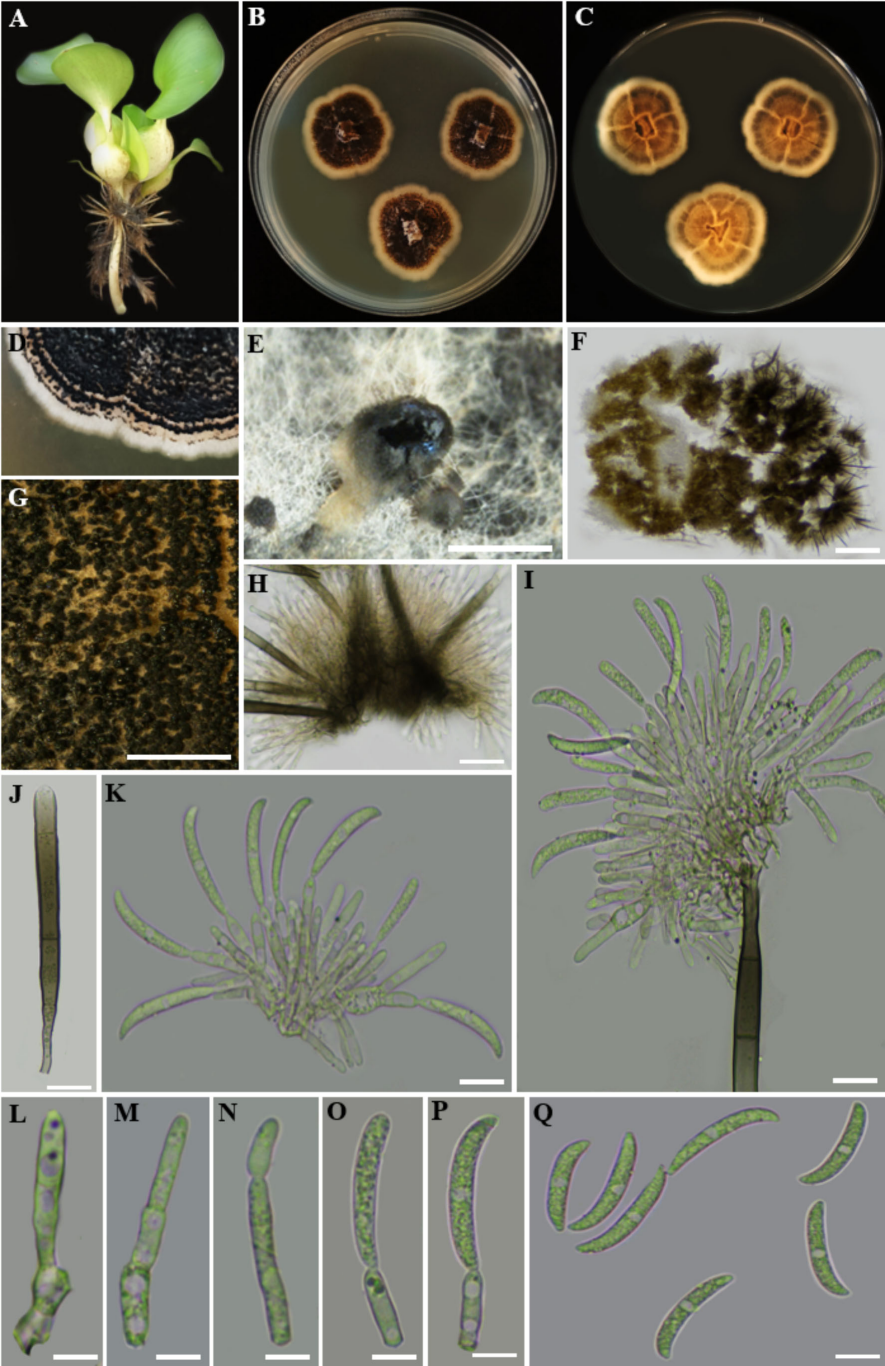
*Colletotrichum truncatum* **(A)** Host freshwater plant leaf of *Eichhornia crassipes* (Water hyacinth). **(B)** Upside of the PDA culture plate (diameter; 9.5 cm). **(C)** Downside of the PDA culture plate (diameter; 9.5 cm) after five days. **(D)** Sporulated culture after ten days. **(E)** Stereo microscopic view of sporulation in culture. **(F)** Stereo microscopic observation of acervuli. **(G, H)** Acervuli. **(J)** Aseta. **(I, K)** Conidiogenesis. **(L–P)** Maturity levels of conidiogenus cells. **(Q)** Conidia. Scale bars: **(E, F)** = 1000 µm, **(G–Q)** = 100 µm.

Index Fungorum Registration Identifier: 280780 (**Figure 12**).

Description: Endophytic of healthy leaf of *Eichhornia crassipes.* Sexual morph: Undetermined. Asexual morph: *Conidiomata* acervular, with conidiophores and setae formed directly on hyphae. *Setae* subhyaline to moderately brown, smooth to verruculose, 2 to 5-septate, cylindrical to conical at base, tapering towards the slightly acute to roundish tip, 4–6 µm diam. *Conidiophores* up to 90 µm long, hyaline to pale brown, septate, densely branched, clustered, *Conidiogenous cells* enteroblastic, phialidic, hyaline to pale brown, cylindrical, 6–20 × 2.5–4 µm, with invisible collarette, periclinal thickening not observed. *Conidia* 20.35–28.39 × 2.23–4 µm ( $\bar{\text{x}}$ = 25 × 3 µm; n = 30), hyaline, cylindric-fusiform, elongated, smooth-walled, aseptate, curved at tapering apex, truncate at base, guttulate with granular content.

Culture characteristics: Colonies on incubating for seven days at 28–30°C in dark on PDA, exhibit a diameter of 1.5–2 cm. The colonies are flat with an entire margin, devoid of aerial mycelium, bluff at surface and covered by olivaceous-grey to iron-grey acervuli. The reverse of the colony is buff to pale olivaceous-grey. Conidia in mass are whitish, buff to pale saffron.

Material examined: SRI LANKA, North Central Province, Mihintale, Mahakanadara tank (8.38683° N, 80.38683° E, 117 m), on healthy leaf of *Eichhornia crassipes* (Water hyacinth), 8 December 2023, Madhara K. Wimalasena, RUFCC2451 (living culture), RUSLH/245 (dried culture as the herbarium specimen).

Notes: De Silva et al. (2019) found that *Colletotrichum* isolates with curved conidia and ITS sequences matching the ex-type of *C. truncatum* were the most common, making up 44% of all isolates. These isolates came from Indonesia, Malaysia, Sri Lanka, and Thailand, while species with straight conidia were identified separately. The remaining 56% were species with straight conidia, mostly from other complexes within the *Colletotrichum* genus. Liu et al. (2022) reported that the *C. truncatum* species complex produces curved conidia. Interestingly, species with curved conidia appear throughout the phylogenetic tree, suggesting this trait evolved multiple times. While ITS is useful for identifying *Colletotrichum* species complexes (Cannon et al., 2012), other loci like *GAPDH*, *ACT*, *CHS-*1, *HIS* 3, and *tub*2 are increasingly used to better define species boundaries, including in the *C. truncatum* complex (Damm et al., 2009, 2014; Liu et al., 2022). See the notes under *Colletotrichum siamense.*

*Dothideomycetes* genera *incertae sedis*

*Neottiosporina* Subram., Proc. Natl. Inst. Sci. India, B 27: 238 (1961)

Index Fungorum Registration Identifier: 9117

Notes: Sutton and Alcorn (1974) revisited the genus *Neottiosporina*, typified by *N. apoda* (Speg.) Subram. (1961), which is characterized by *pycnidia* that are solitary, dark brown, globose to subglobose, thin-walled, and ostiolate; *conidiogenous cells* are holoblastic, solitary, hyaline, determinate, and originate from the inner wall of the pycnidium. The *conidia* are acrogenous, solitary, hyaline, multiseptate, smooth-walled, cylindrical to cymbiform, obtuse at apex, and truncate at base. The genus comprises ten species *viz*., *N. apoda* (Speg.) Subram (Sutton and Alcorn, 1974), *N. ashworthiae* (From Scleria: Queensland *fide* Tan and Shivas, 2022), *N. asymmetrica* (on *Themeda australis fide* Sutton and Alcorn, 1974), *N. australiensis* (on *Phragmites australis fide* Sutton and Alcorn, 1974), *N. clavata* (on *Phragmites australis fide* Sutton, 1981), *N. masonii* (on *Pinus caribaea fide* Sutton and Sarbhoy, 1976), *N. cylindrica* (on *Cyperus brevifolius fide* Sutton and Alcorn, 1985), *N. paspali* (Sutton and Alcorn, 1974), *N. phragmiticola* (in Ethiopia, Sudan, and Uganda *fide* (Nag Raj, 1993), and *N. sorghicola* in China (Sutton and Wu, 1995). In our study of endophytic fungi inhabiting aquatic plant species, we isolated a novel taxon of *Neottiosporina.*

***Neottiosporina mihintaleensis*** Wimalasena, Wijayaw. & Bamunuarachchige sp. nov.

**Figure 13:**
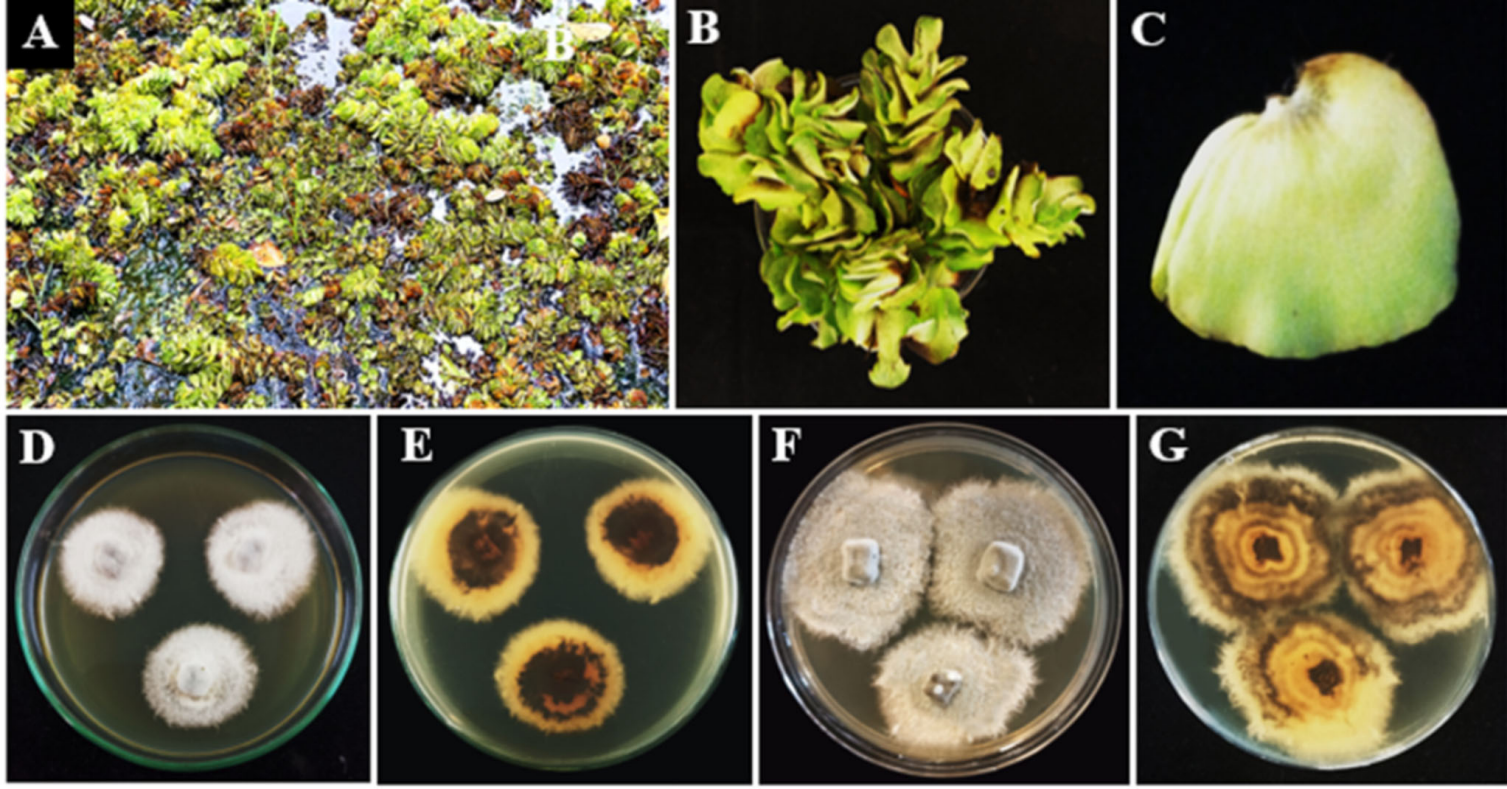
*Neottiosporina mihintaleensis* sp. nov. **(A–C)** Host freshwater plant leaf of *Salvinia molesta* (Giant Salvinia). **(D)** Upside of the PDA culture plate (diameter; 9.5 cm). **(E)** Downside of the PDA culture plate (diameter; 9.5 cm) after five days. **(F)** Upside of the PDA culture plate (diameter; 9.5 cm). **(G)** Downside of the PDA culture plate (diameter; 9.5 cm) after ten days.

Index Fungorum Registration Identifier: IF902502 (**Figure 13**).

Etymology: The name is derived from the locality from where the fungus was collected.

Holotype: RUSLH/241

Description: Endophytic of healthy leaf of *Salvinia molesta.* Sexual and asexual morphs undetermined. Despite efforts to induce the asexual morph using sporulation techniques described in the materials and methods section (2.1), the fungal cultures did not sporulate. Thus, we conclude it is sterile mycelia.

Culture characteristics: Colonies grown on PDA at 28–30°C in a 2 cm Petri dish over a two-week period show that the superficial mycelium is abundant and dark grey at the centre, with white patches towards the periphery. The immersed mycelium appears pale to medium brown with an irregular margin. On the reverse side, the colony displays a centre ranging from yellowish-brown to dark brown, transitioning to a yellowish-white colour at the margin.

Material examined: SRI LANKA, North Central Province, Mihintale, Mahakanadara tank (8.38683° N, 80.38683° E, 117 m), on healthy leaf of *Salvinia molesta* (Giant Salvinia), 10 December 2023, Madhara K. Wimalasena, RUSLH/241 (holotype as the dry culture), RUFCC2454 (ex-type); *ibid* RUFCC2461 (living culture).

Notes: *Neottiosporina mihintaleensis* sp. nov. is a newly identified species within the genus *Neottiosporina*, discovered in freshwater environments in Sri Lanka. Phylogenetic analysis indicates that it is closely related to *N. cylindrica* and *N. ashworthiae* (**Figure 7**). *Neottiosporina cylindrica* produces cylindrical to slightly clavate conidia (Sutton and Alcorn, 1985; Li et al., 2020) whereas *N. ashworthiae* has not reported its micromorphological characters. However, *Neottiosporina mihintaleensis* did not produce asexual morph in culture, despite the use of sporulation techniques (see sporulation techniques in materials and methods 2.1), making it impossible to compare its morphological features along with phylogenetically related species. Hence, we introduce *Neottiosporina mihintaleensis* as sterile mycelia.

### 3.3 Qualitative enzymatic assay for extracellular enzymes production by endophytic fungi

This study shows that fungal isolates can produce amylase, cellulase, and laccase enzymes. This enhances our understanding of their ecological roles and opens avenues for future biotechnological applications in diverse industries, thereby addressing the ongoing demand for enzymatic solutions in global markets.

#### 3.3.1 Production of amylase enzyme by fungal isolates

The amylase activity shown by these endophytes can help break down starch when plants start to age (Mahfooz et al., 2017). As biotechnology advances, the significance of amylases in the production of various commodities, such as food and starch-based products, continues to grow. Given the widespread utilization of these enzymes across numerous industries, there exists a persistently high demand for amylases (Khokhar et al., 2011; Bilal and Iqbal, 2019; Patil et al., 2021). As a result, there is an ongoing search for new microbial strains that can produce these enzymes (Khokhar et al., 2011; Patil et al., 2021). The emergence of these newly identified fungal isolates suggests promising prospects for large-scale amylase production.

**Figure 14:**
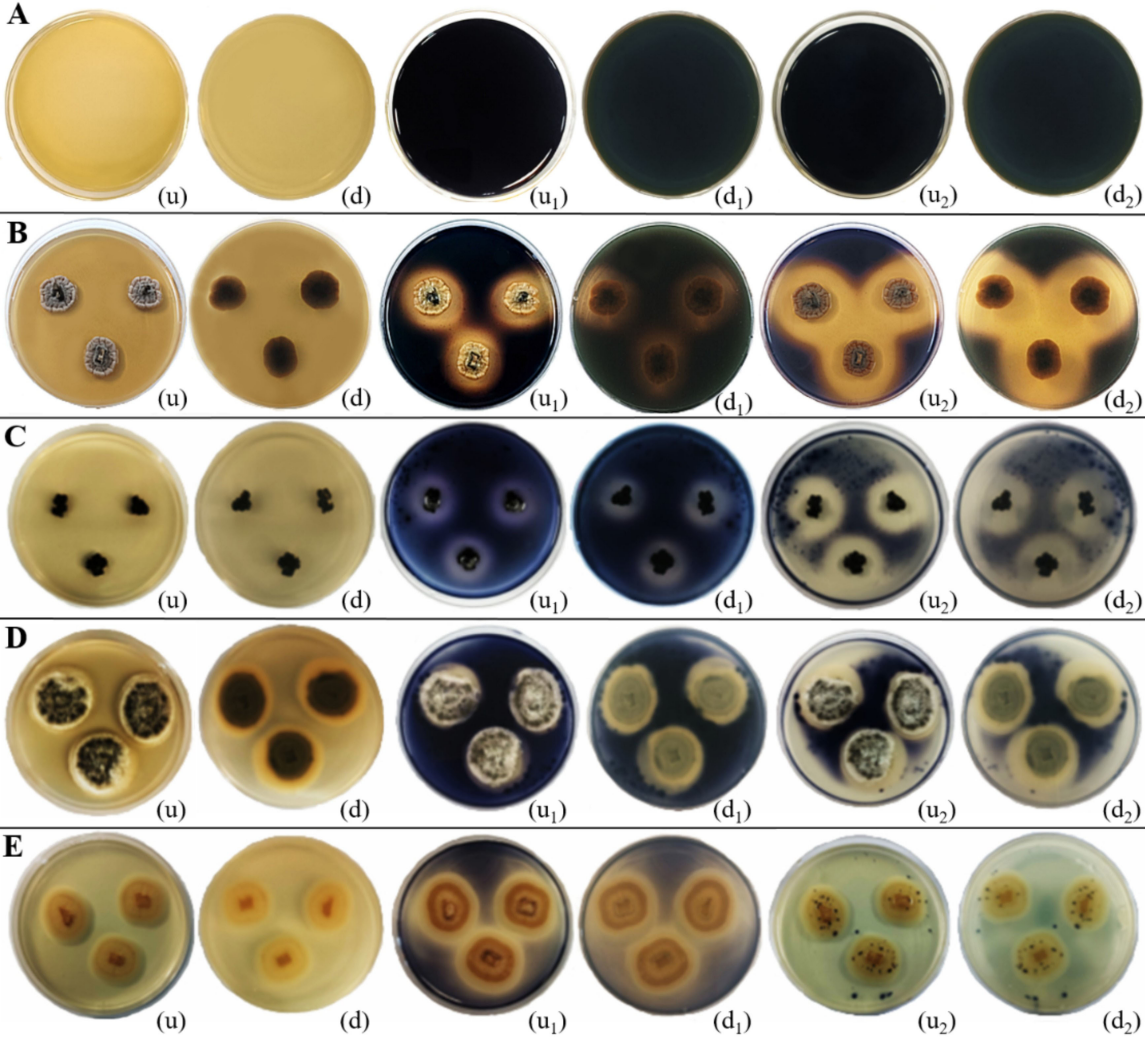
Amylase enzymatic activity of endophytic fungi isolated from freshwater plants. **(A)** Negative control. **(B)** *Ectophoma salviniae* sp. nov. **(C)** *Phyllosticta capitalensis.* **(D)** *Colletotrichum siamense.* **(E)** *Chaetomella raphigera.* (u, d) Upside and downside of the plate before adding 1–2 mL of iodine solution. (u_1_, d_1_) Upside and downside of the plate after adding 1–2 mL of iodine solution for 15 minutes (u_2_, d_2_) Upside and downside of the plate after adding 1–2 mL of iodine solution for 30 minutes, respectively. Amylase enzymatic activity was indicated by the clear zone appearance of the fungal colony on PDA media supplemented with 1% starch. **(B–E)** Positive control.

To assess amylase production, the positive control contained fungal endophytes cultured on PDA media supplemented with 1% soluble starch. Following a seven-day incubation period at 28–30°C (range of the room temperature), 1–2 mL of iodine solution was applied to flood the culture plates, resulting in a blue-black coloration. Observations were recorded at 15 minute and 30 minute intervals. A change from blue-black to a colorless medium indicated the presence of amylase activity, as the enzyme catalyzed the hydrolysis of starch. Among the isolates, *Ectophoma salviniae* sp. nov. exhibited the highest amylase production, forming a clear zone with a diameter of 2.5 cm around the fungal colony and decolorizing the medium completely within 10 minutes, while *Phyllosticta capitalensis* produced a clear zone of 1.5 cm in diameter around its colony after 15 minutes. Other fungal isolates also displayed amylase production, with discernible effects after 15 minutes. All endophyte isolates, except *Neottiosporina mihintaleensis* sp. nov. and *C. truncatum*, have exhibited the ability to produce extracellular amylase, underscoring their notable enzymatic capabilities (**Table 6**; **Figure 14**). Prior research has documented amylase activity in species like *Phyllosticta* spp. (Wikee et al., 2017; Reyes et al., 2021). and *Colletotrichum* spp. (Prajapati et al., 2013; Armesto et al., 2020; da Silva et al., 2021). The comparison with the negative control, which consisted of PDA media supplemented with 1% soluble starch and without the inoculation of endophytic fungi, involved a seven-day incubation period at 28–30°C. Following this incubation, 1–2 mL of iodine solution was applied to flood the culture plates, resulting in a blue-black coloration. Observations were recorded at 15 minute and 30minute intervals; however, no color change occurred, and the blue-black coloration remained (**Figure 14**).

**Table 6:** The ability for extracellular enzymes production by endophytic fungi isolated from freshwater plants.

Endophytic fungal strains	Extracellular enzymes production		
	Amylase	Cellulase	Laccase
*Chaetomella raphigera*	+	+	–
*Colletotrichum siamense*	+	+	–
*C. truncatum*	–	+	+
*Ectophoma salviniae* sp. nov.	+	+	+
*Phyllosticta capitalensis*	+	+	+
*Neottiosporina mihintaleensis* sp. nov.	–	–	+

“+” denotes the ability of fungi to produce extracellular enzymes, while “–” represents the inability of fungi to produce extracellular enzymes.

#### 3.3.2 Production of cellulase enzyme by fungal isolates

Cellulase has significant applications across various industries, making it a highly researched enzyme in academic and industrial settings. It is particularly valuable in the pulp and paper, textile industry, bio-ethanol production, wine and brewery sectors, food industry, extraction of pigments and bioactive compounds, pharmaceutical industries, and waste management (Srivastava et al., 2018; Dhevagi et al., 2021; Ejaz et al., 2021; Maravi and Kumar, 2021; Singh et al., 2021; Łubek-Nguyen et al., 2022). Due to its broad utility, cellulase is in high demand, accounting for approximately 20% of the global enzyme market (Srivastava et al., 2015; Singh et al., 2021). Fungal cellulase enzymes are particularly effective in breaking down the cellulose component of lignocellulosic materials into hexose sugars, making fungi good producers of cellulase enzymes among microorganisms (Singh et al., 2021).

**Figure 15:**
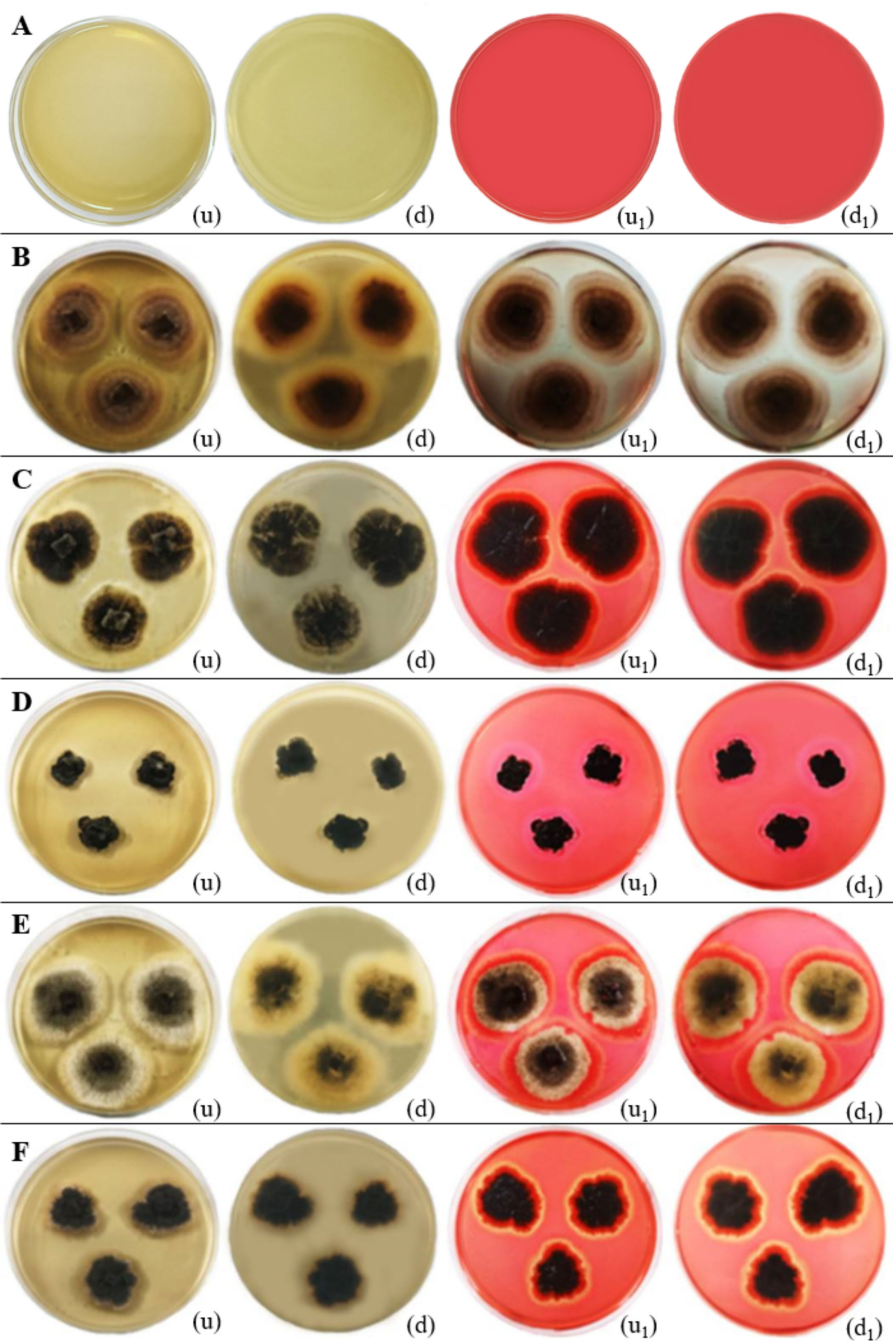
Cellulase enzymatic activity of endophytic fungi isolated from freshwater plants. **(A)** Negative control. **(B)** *Chaetomella raphigera.* **(C)** *Colletotrichum truncatum.* **(D)** *Phyllosticta capitalensis.* **(E)** *Colletotrichum siamense.* **(F)** *Ectophoma salviniae* sp. nov. (u, d) Upside and downside of the plate before adding 0.1% (w/v) Congo red solution respectively. (u_1_, d_1_) Upside and downside of the plate after adding 0.1% (w/v) Congo red solution respectively. Cellulase enzymatic activity was indicated by the clear zone appearance of the fungal colony on PDA media. **(B–F)** Positive control.

In this study, the positive control consisted of endophytic fungi inoculated into PDA media supplemented with 0.5% (w/v) sodium carboxymethyl cellulose and incubated for five days at 28–30°C. Following incubation, 0.1% (w/v) Congo red was applied, followed by 1M NaCl for 5 minutes to visualize the enzymatic activity (clear halo around the colonies). *Chaetomella raphigera* exhibited the highest cellulase enzyme production, as evidenced by the red media turning colorless with a clear halo. *Phyllosticta capitalensis* formed clear halos around each colony, indicating significant cellulase enzyme production, second only to *Chaetomella raphigera.* The other isolates (*Colletotrichum truncatum, C. siamense*, and *Ectophoma salviniae* sp. nov) exhibited only minimal cellulase enzyme production on solid media, whereas *Neottiosporina mihintaleensis* sp. nov. displayed no cellulase enzyme activity (**Table 6**; **Figure 15**). In the comparison between the positive and negative controls, the negative control consisted of PDA media supplemented with 0.5% (w/v) sodium carboxymethyl cellulose, without the inoculation of endophytic fungi, and incubated for five days at 28–30°C. After incubation, 0.1% (w/v) Congo red was applied, followed by a 5-minute treatment with 1M NaCl. No enzymatic activity (clear halo) was observed, leaving only the Congo red stain visible on the plates (**Figure 15**).

Previously, Kao et al. (2019) and Singh et al. (2023) have found that *Chaetomella* sp. exhibits a high capacity for producing glucose-tolerant cellulase enzymes. Similarly, Amirita et al. (2012); Yopi et al. (2017), and Wikee et al. (2017) have highlighted the significant potential of *Phyllosticta* sp. for cellulase enzyme production.

#### 3.3.3 Production of laccase enzyme by fungal isolates

Laccases, classified as blue multicopper oxidases, catalyze the one-electron oxidation of a wide range of substrates and play a crucial role in lignin degradation (Abdel-Hamid et al., 2013; Viswanath et al., 2014; Singh and Gupta, 2020; Kyomuhimbo and Brink, 2023; Sharma et al., 2024). These enzymes are extensively used in various industries, including industrial effluent decolorization and detoxification, wastewater treatment, paper and pulp production, textiles, xenobiotic degradation, bioremediation, and as biosensors, owing to their key role in the breakdown of lignin and phenolic compounds (Shraddha et al., 2011; Viswanath et al., 2014; Singh and Gupta, 2020; Khatami et al., 2022). Laccase have been identified in approximately 60 fungal strains from the genera *Ascomycetes, Deuteromycetes*, and *Basidiomycetes* (Leonowicz et al., 2001; Albu et al., 2019; Abo Nahas et al., 2021; Mahuri et al., 2023). Fungal laccases are categorized into two types: true laccase and false laccase (De Jesus et al., 2009; Mahuri et al., 2023). True laccases can oxidize phenols and aminophenols but cannot oxidize the amino acid residue tyrosine. On the other hand, false laccases can oxidize tyrosine (De Jesus et al., 2009; Chauhan et al., 2017; Jayaram et al., 2023; Mahuri et al., 2023). Jayaram et al. (2023) highlighted that laccase production by fungal endophytes is a promising area of research due to its potential industrial applications, such as bioremediation and detoxification of pollutants.

**Figure 16:**
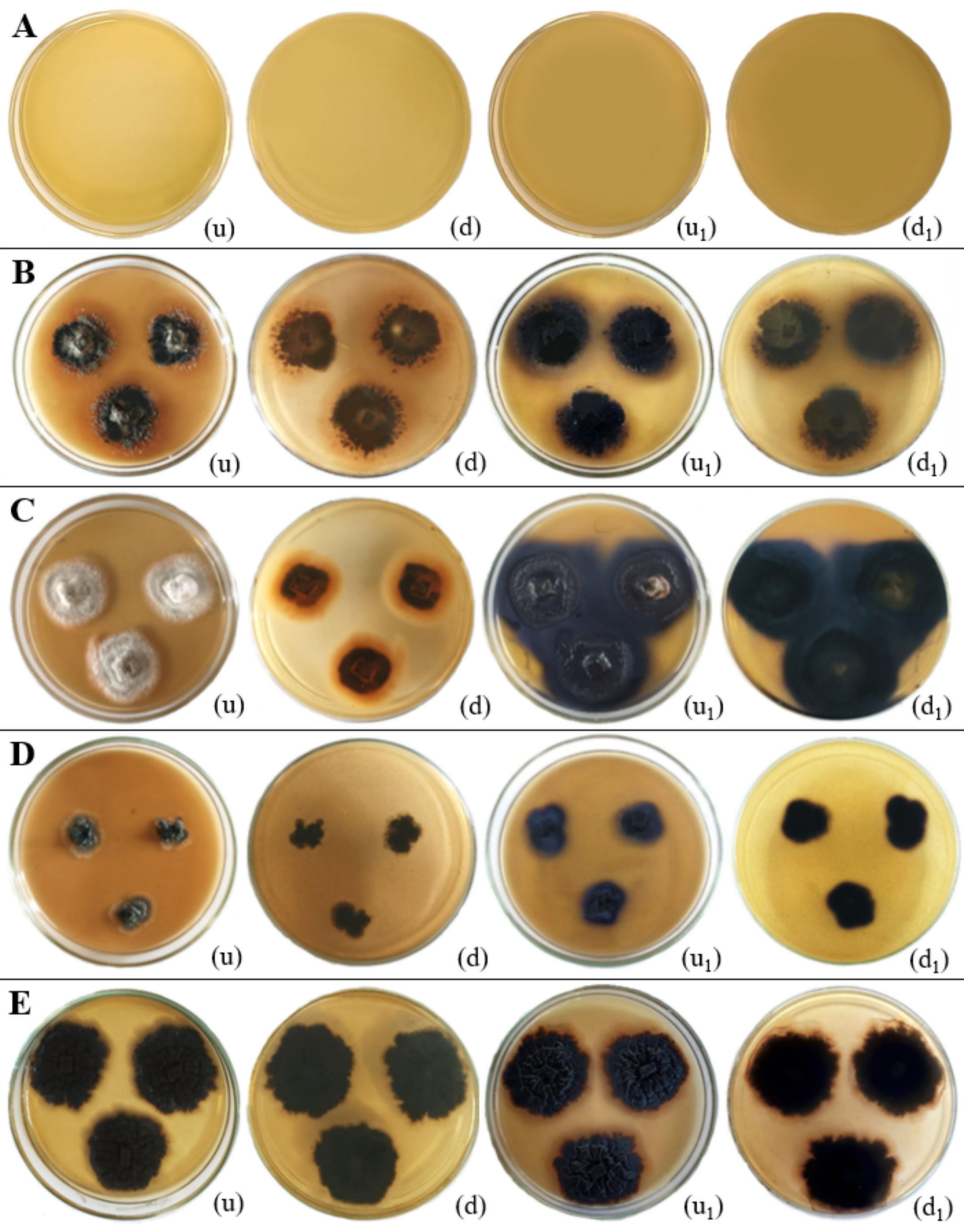
Laccase enzymatic activity of endophytic fungi isolated from freshwater plants. **(A)** Negative control. **(B)** *Colletotrichum truncatum.* **(C)** *Neottiosporina mihintaleensis* sp. nov. **(D)** *Phyllosticta capitalensis.* **(E)** *Ectophoma salviniae* sp. nov. (u, d) Upside and downside of the plate before adding 0.1M 1-Naphthol respectively. (u_1_, d_1_) Upside and downside of the plate after adding after adding 0.1M 1-Naphthol respectively. Laccase enzymatic activity was indicated by the blue purple colour appearance of the fungal colony on PDA media. **(B–E)** Positive control.

As per the findings of this study, upon comparing the negative and positive controls in the laccase assay, no color change (blue-purple coloration) was observed in the negative control after the addition of 1-Naphthol solution droplets and 24 hours of incubation at 28–30°C (**Figure 16**). In the positive control, the addition of 1-Naphthol solution droplets to each colony of endophyte isolates, followed by subsequent incubation for 24 hours at 28–30°C, resulted in a blue-purple coloration at the edges of the colonies, indicating the presence of laccase enzymes. This reaction was observed in *Colletotrichum truncatum*, *Ectophoma salviniae*, *Neottiosporina mihintaleensis*, and *Phyllosticta capitalensis*. Among these, *Neottiosporina mihintaleensis* exhibited the highest laccase production, with a 3.5 cm diameter blue-purple circle around the colony. *Phyllosticta capitalensis* showed the second-highest laccase production, with a 1 cm diameter blue-purple circle around the colony, compared to its appearance before applying 1-Naphthol droplets. In contrast, *Chaetomella raphigera*, and *C. siamense* did not exhibit laccase enzyme production in this qualitative assay (**Table 6**; **Figure 16**).

This research has shown the novel discovery of laccase production in *N. mihintaleensis* and *E. salviniae*. These findings contribute to an expanding understanding of fungal laccases. Notably, prior studies have extensively documented laccase production capabilities in *Phyllosticta* spp. (Wikee et al., 2017; Shankar Naik et al., 2019), *C. truncatum* (Levin et al., 2007; Núñez et al., 2023).

## 4 Discussion

### 4.1 Exploration of endophytic fungi in Sri Lanka’s freshwater environments

#### 4.1.1 Current status and research gaps on endophytic fungal study in Sri Lanka

Currently, only around 3,000 fungal species are known in Sri Lanka, with an estimated 31,000 plant-associated species still to be described (Adikaram and Yakandawala, 2020; Wijayawardene et al., 2022b). Several of the known fungi in the island nation remain unpublished and have not been sufficiently studied or documented (Adikaram and Yakandawala, 2020; Karunarathna et al., 2022; Wijayawardene et al., 2022b, 2023; Wimalasena et al., 2024). In the Sri Lankan context, particularly regarding endophytic fungi, many studies have focused on terrestrial plants (e.g (Alwis et al., 2021; Pathmanathan et al., 2022; Koshila et al., 2023; Undugoda et al., 2023). Studies on endophytic fungi associated with aquatic plants in Sri Lanka are relatively scarce (Rajagopal et al., 2018; Ravimannan and Sepali, 2020; Ekanayake et al., 2021). Hitherto, the identification of endophytic fungi in freshwater plants was based largely on morphological characters. For instance, Hettiarachchi et al. (1983) reported 15 fungi (*Alternaria* sp., *Cephalosporium* sp., *Cercospora piaropi, Curvularia tuberculata, Fusarium* sp., *Idriella lunata, Mucor* sp., *Myrothecium roridum, Neurospora* sp., *Penicillium oxalicum, Phaeotrichoconis crotalariae*, and *Septofusidium elegantulum*) isolated from *Eichhornia crassipes*, with identification based only on morphologal charcteristics. In some of the Sri Lankan studies, endophytic fungal identification was based on a single gene locus, which is inadequate for accurate identification. For example, Dissanayake et al. (2014, 2016) identified *Chaetomium globosum* from healthy *Nymphaea nouchali* using only the ITS locus.

#### 4.1.2 Identification of endophytic fungi in Sri Lankan freshwater habitats: potential for novel species discovery

Sri Lanka harbors over 370 aquatic and wetland plant species, with 12% being unique to the country (Yakandawala, 2012; Bambaranda et al., 2024). These endemic plants serve as essential habitats for fungi, including freshwater fungi and endophytic species that have adapted to unique environmental conditions (Ratnaweera, 2019; Wimalasena et al., 2024). Wimalasena et al. (2024), highlighted that these habitats offer substantial potential for the collection, identification, and utilization of endophytic fungi found in freshwater plants.

This study focused on isolation of freshwater endophytic fungi in three lentic freshwater habitats (Iluppukanniya tank, Mahakanadara tank, and Mihintale tank) located in Mihintale area within the Anuradhapura district. Using polyphasic approaches, six endophytic fungi were isolated including two novel taxa, *Ectophoma salviniae* sp. nov. and *Neottiosporina mihintaleensis* sp. nov. These fungal species were identified in their endophytic life modes, occurring within healthy freshwater plant tissues, particularly in healthy leaves, isolated by a culture-dependent method. In this study, the invasive plant species *Eichhornia crassipes* (Ayanda et al., 2020; Maulidyna et al., 2021; Bayu et al., 2024) provided a wider range of host substrates for fungi compared to other freshwater plants such as *Salvinia* and *Nymphaea*, highlighting its value for biodiversity. Hence, expanding such studies to cover more freshwater habitats could lead to the identification of additional novel species, contributing significantly to the field of mycology and biotechnology worldwide.

### 4.2 Reference cultures of pathologically important taxa, *Colletotrichum siamense*, *C. truncatum* and *Ectophoma* sp. in Sri Lanka

Precise identification of fungi is an important step in taxonomy. DNA sequence analyses and morphological characters play an important role in modern taxonomy which aids in identifying species and providing their classification (Wijayawardene et al., 2023). A large number of species originally described from Sri Lanka lack sequence data and were identified based on only morphological characteristics (Wijayawardene et al., 2022b). Nevertheless, delineating species boundaries of species complexes of specious genera would depend only on DNA sequence data analyses.

Adikaram and Yakandawala (2020) listed pathologically important *Colletotrichum* species in Sri Lanka, including *C. siamense* and *C. truncatum.* However, either *C. siamense* or *C. truncatum* have not been reported as a pathogenic species from aquatic plants. **Table 7** lists the studies that provided phylogenetic identifications of *C. siamense* and *C. truncatum.*

**Table 7:** Studies provided phylogenetic analyses for *Colletotrichum siamense* and *C. truncatum* species in Sri Lanka (RUFCC-Rajarata University Fungal Culture Collection, UOM-University of Melbourne culture collection, Victoria, Australia, UPBT-University of Peradeniya, Department of Biotechnology, USJCC-University of Sri Jayewardenepura Culture Collection, Department of Botany, University of Sri Jayewardenepura, Nugegoda, Sri Lanka).

Species name	Host	Life mode	Gene regions	Culture collections	References
*C. truncatum*	*Capsicum annuum*	Pathogen	ITS, *tub*2, *GADPH*, *CHS*-1, *HIS*3 and *ACT*	UOM	(De Silva et al., 2019)
	*Eichhornia crassipes*	Endophyte	ITS, *tub*2, *ACT*, *CHS*-1 and *GADPH*	RUFCC	This study
*C. siamense*	*Capsicum annuum*	Pathogen	ITS, *tub*2, *GADPH*, *CHS*-1, *HIS*3 and *ACT*	UOM	(De Silva et al., 2019)
	*Persea Americana*	Pathogen	ITS, *tub*2, and *GADPH*	UPBT	(Dissanayake et al., 2021)
	*Allium cepa*	Pathogen	ITS, *GADPH* and *tub*2	USJCC	(Herath et al., 2021)
	*Eichhornia crassipes*	Endophyte	ITS, *tub*2, *ACT*, *CHS*-1 and *GADPH*	RUCC	This study

In a previous study, Dissanayake et al. (2016) reported *C. siamense* from *Nymphaea nouchali* but they used only the ITS region to identify the taxon. Use of one locus is not recommended for *Colletotrichum* thus, we used ITS, *tub*2, *ACT*, *CHS* **-**1 and *GADPH* regions in our phylogenetic analyses following Armand et al. (2023) and Armand and Jayawardena (2024). We have not observed any disease symptoms in the leaves of *Eichhornia crassipes.* Hence, it is concluded that both *C. siamense* and *C. truncatum* are endophytic species of *Eichhornia crassipes.* This is the first study that provided multi-locus phylogenetic evidence to identify two pathologically important (but endophytic in this study) *Colletotrichum* species (e.g., *C. siamense* and *C. truncatum*) in aquatic habitats. It is important to maintain the reference living cultures of both species; thus, it has been deposited at the Rajarata University Culture Collection.

*Ectophoma* species have been reported as important plant pathogens. *Ectophoma multirostrata*, the type species of *Ectophoma* (Valenzuela-Lopez et al., 2018), has been originally reported as a soil-inhabiting fungi in India (as *Sphaeronaema multirostratum fide* (Mathur and Thirumalachar, 1959). Later, this species was reported as a pathogen of different plants worldwide (e.g (Aveskamp et al., 2010; Valenzuela-Lopez et al., 2018; Chobe et al., 2020; Ahmadpour et al., 2021; Kularathnage et al., 2023). Lee et al. (2022) reported *Ectophoma multirostrata* as a pathogenic agent infecting the aquatic plant water spinach (*Ipomoea aquatica*) in Korea. *Ectophoma myriophyllana* Huang Y. and Yu Z. F. was recently introduced as an epiphyte of leaves of *Myriophyllum spicatum* (Chen et al., 2023). Our novel species, *Ectophoma salviniae* did not cause any diseased symptoms on the leaves of *Salvinia minima* and thus, we conclude it is an endophytic taxon inhabiting the host. As far as we know, this is the first report of *Ectophoma* species from *Salvinia* species in Sri Lanka (Farr and Rossman, 2024). We have not observed *Ipomoea aquatica* (which was affected by *Ectophoma multirostrata*) in the same aquatic environment, and the distribution of *Ectophoma salviniae* sp. nov. is unknown. Future studies would be essential to recognise the potential host jumping and life mode switching of *Ectophoma salviniae* sp. nov. and its impact on *Ipomoea aquatica* since it is a widely-used leafy vegetable in Sri Lanka.

### 4.3 The possibility of endophytic fungi being used as mycoherbicides against invasive weed management in wetland environments

#### 4.3.1 Threat of invasive aquatic plants

Invasive plant species pose a major threat to natural ecosystems by reducing biological diversity (Rodríguez-Merino, 2023; Xiong et al., 2023). Over the past few decades, the spread of aquatic alien plant species in the lentic water bodies of Sri Lanka has created significant ecological, environmental, and economic problems (Dissanayake, 2020; Kariyawasam et al., 2021). Thus, fungi can be used as mycoherbicides for a more effective solution to eradicate invasive aquatic plant species due to their pathogenic activity and host-specific targeting.

#### 4.3.2 Application of fungi as mycoherbicides

Bioherbicides are biological products used to control weed species and are typically formulated using microbiological agents, especially fungi, and are often referred to as mycoherbicides (Golijan et al., 2023; Ravlić and Baličević, 2014). The concept of mycoherbicides emerged during the 1980s and 1990s, as documented by TeBeest and Templeton (1985); Templeton (1987); Templeton (1992) and Wall et al. (1992). Mycoherbicides are considered environmentally friendly alternatives to chemical herbicides because they are harmless to the environment, eco-friendly, and specifically target certain types of plants (Chakraborty and Ray, 2021; Hasan et al., 2021; Keshamma, 2022). The efficiency of fungi in weed management is exemplified by the reported potential of *Colletotrichum* species (**Table 8**). *Colletotrichum siamense* and *C. truncatum* have previously been identified as pathogenic fungi across various plant species. According to this study, these *Colletotrichum* spp. may demonstrate potential for managing the invasive weed *Eichhornia crassipes* in Sri Lanka.

**Table 8:** The potential application of *Colletotrichum* species in weed management.

*Colletotrichum* spp.	Target weeds	References
*C. gloeosporioides* BWH-1	*Alopecurus aequalis, Amaranthus retroflexus, Ageratum conyzoides, Bidens pilosa, Capsella bursa-pastoris, Celosia argentea, Echinochloa crusgalli, and Mikania micrantha*	(Xu et al., 2019)
*C. dematium*	*Parthenium hysterophorus*	(Singh et al., 2010)
*C. gloeosporioides*	Russian thistle, Tumblewee	(Berner et al., 2009)
*C. graminicola*	*Echinochloa* sp.	(Yang et al., 2007)
*C. lini*	*Convolvulus arvensis*	(Damm et al., 2014; Tunali et al., 2008, 2009)

## 5 Conclusion

This study identified the culturable mycobiota in three lentic freshwater habitats located in Mihintale, within the Anuradhapura District of Sri Lanka, revealing a rich fungal diversity. Through identification, six endophytic fungal species were found, including two novel endophytic fungal species: *Ectophoma salviniae* sp. nov. and *Neottiosporina mihintaleensis* sp. nov., recorded on the freshwater plant *Salvinia*. The identification was confirmed using a polyphasic approach. The next step involved qualitatively assessing the extracellular enzymatic potentials of these endophytic isolates. *Ectophoma salviniae* sp. nov. exhibited the highest amylase production, *Chaetomella raphigera* showed the highest cellulase enzyme production, and *Neottiosporina mihintaleensis* sp. nov. demonstrated the highest laccase production, offering novel insights for future biotechnological applications. Besides, this study discussed the potential of fungi as mycoherbicides for managing invasive freshwater weeds.

## Data Availability

The datasets presented in this study can be found in online repositories. The names of the repository/repositories and accession number(s) can be found below: https://www.ncbi.nlm.nih.gov/nuccore/genbank/, ITS: PP989214, PP989215, PP989216, PP989217, PP989218, PP989219, PP989220, PP989221, PP989222; LSU: PP989223, PP989224, PP989225, PP989226, PP989227; SSU: PP989228, PP989229, PP989230; *GAPDH*: PQ014240, PQ014241, PQ014242, PQ014243; *CHS-1*: PQ014237, PQ014238, PQ014239; *ACT*: PQ014233, PQ014234, PQ014235, PQ014236; *tub*2: PQ014246, PQ014247, PQ014248; *rpb*2: PQ014244, PQ014245; *tef1*-α: PQ014249.
