# Supplementary material for: Ectophoma salviniae sp. nov., Neottiosporina mihintaleensis sp. nov. and four other endophytes associated with aquatic plants from Sri Lanka and their extracellular enzymatic potential
Source: Front Cell Infect Microbiol. 2025 Jan 8;14:1475114. doi: 10.3389/fcimb.2024.1475114 (PMC11750795; doi:10.3389/fcimb.2024.1475114)
Supplement: Supplementary file 1 [file DataSheet1.pdf]

GenBank accession numbers of endophytic fungal species in the manuscript

The names of the repository/repositories and accession number(s) can be found below: https://www.ncbi.nlm.nih.gov/nuccore/genbank/, ITS: PP989214, PP989215, PP989216, PP989217, PP989218, PP989219, PP989220, PP989221, PP989222; LSU: PP989223, PP989224, PP989225, PP989226, PP989227; SSU: PP989228, PP989229, PP989230; *GAPDH*: PQ014240, PQ014241, PQ014242, PQ014243; *CHS*-1: PQ014237, PQ014238, PQ014239; *ACT*: PQ014233, PQ014234, PQ014235, PQ014236; *tub*2: PQ014246, PQ014247, PQ014248; *rpb*2: PQ014244, PQ014245; *tef*1-α: PQ014249

Taxa	Voucher/Strain	GenBank accession number				
		**ITS**	**LSU**	**SSU**		
Details of sequences used for *Chaetomella raphigera* phylogenetic analyses						
Chaetomella raphigera	RUFCC2453	PP989214	PP989223	PP989228		
Details of sequences used for *Colletotrichum* phylogenetic analyses						
Taxa	Voucher/Strain	GenBank accession number				
		**ITS**	***GAPDH***	***CHS*-1**	***ACT***	***tub*2**
Colletotrichum siamense	RUFCC2455	PP989215	PQ014240	PQ014237	PQ014233	PQ014246
Colletotrichum siamense	RUFCC2457	PP989216	PQ014241	PQ014238	PQ014234	PQ014247
Colletotrichum truncatum	RUFCC2451	PP989217	PQ014242	PQ014239	PQ014235	PQ014248
Details of sequences used for *Ectophoma* phylogenetic analyses						
Taxa	Voucher/Strain	GenBank accession number				
		**ITS**	**LSU**	***rpb*2**		
Ectophoma salviniae	RUFCC2458T	PP989218	PP989224	PQ014244		
Ectophoma salviniae	RUFCC2462	PP989219	PP989225	PQ014245		
Details of sequences used for *Neottiosporina* phylogenetic analyses						
Taxa	Voucher/Strain	GenBank accession number				
		**ITS**	**LSU**	**SSU**		
Neottiosporina mihintaleensis	RUFCC2454T	PP989220	PP989226	PP989229		
Neottiosporina mihintaleensis	RUFCC2461	PP989221	PP989227	PP989230		

Details of sequences used for *Phyllosticta capitalensis* phylogenetic analyses

Taxa	Voucher/Strain	GenBank accession number				
		ITS	*tef*1-α	*ACT*	*GADPH*	
Phyllosticta capitalensis	RUFCC2452	PP989222	PQ014249	PQ014236	PQ014243	
