# Supplementary material for: Ectophoma salviniae sp. nov., Neottiosporina mihintaleensis sp. nov. and four other endophytes associated with aquatic plants from Sri Lanka and their extracellular enzymatic potential
Source: Front Cell Infect Microbiol. 2025 Jan 8;14:1475114. doi: 10.3389/fcimb.2024.1475114 (PMC11750795; doi:10.3389/fcimb.2024.1475114)
Supplement: Supplementary file 2 [file Table1.doc]

**Supplementary table 1:** Details of sequences used for *Chaetomella* phylogenetic analyses

| **Taxa** | **Voucher/Strain** | **GenBank accession number** | | |
| --- | --- | --- | --- | --- |
|  |  | **ITS** | **LSU** | **SSU** |
| *Chaetomella acutiseta* | AFTOL-ID 270 | AY004230 | AY544679 | AY544728 |
| *C. endophytica* | SDBR-CMU300^T^ | MG406985 | MG406984 | MG406986 |
| *C. oblonga* | CBS 113709^T^ | MH862940 | N/A | N/A |
| *C. oblonga* | BPI 843552 | AY487079 | AY487080 | AY487081 |
| *C. pseudocircinoseta* | CBS 145549^T^ | MK876379 | MK876418 | N/A |
| *C. raphigera* | CBS 120.57 ^T^ | MH857671 | MH869210 | N/A |
| *C. raphigera* | BPI 843551 | AY487085 | AY487086 | AY487087 |
| *C. raphigera* | BPI 843541 | AY487076 | AY487077 | AY487078 |
| *C. raphigera* | JCM 9995 | LC228653 | LC228710 | N/A |
| ***C. raphigera*** | **RUFCC2453** | **PP989214** | **PP989223** | **PP989228** |
| *C. zambiensis* | CBS 137978^T^ | KJ869130 | KJ869187 | N/A |
| *Pilidium acerinum* | CBS 736.68^T^ | NR119500 | AY487092 | AY487093 |
| *P. acerinum* | PBI 843554 | AY487088 | AY487089 | AY487090 |
| *P. concavum* | BPI 1107274 | AY487097 | AY487098 | AY487099 |
| *P. eucalyptorum* | CPC 26594^T^ | KT950854 | KT950868 | N/A |
| *P. lythri* | CCTU: PN6 | KX639607 | KX639613 | N/A |
| *P. pseudoconcavum* | CPC 21642^T^ | KF777184 | KF777236 | N/A |
| *Sphaerographium nyssicola* | CBS 128284^T^ | NR119916 | N/A | N/A |
| *S. nyssicola* | AR4654 | HQ338472 | N/A | N/A |
| *Synchaetomella acerina* | DAOM242271^T^ | NR111811 | NG042747 | JX989832 |
| *Xeropilidium dennisii* | KL251T | LT158441 | KX090824 | KX090876 |
| *X. dennisii* | KL159 | LT158422 | KX090807 | KX090859 |
| *Hymenoscyphus fructigenus* | CBS 186.47^T^ | EU940233 | EU940157 | EU940081 |
| *H. scutula* | CBS 101.66^T^ | AY789432 | AY789431 | AY789430 |
