# Supplementary material for: Ectophoma salviniae sp. nov., Neottiosporina mihintaleensis sp. nov. and four other endophytes associated with aquatic plants from Sri Lanka and their extracellular enzymatic potential
Source: Front Cell Infect Microbiol. 2025 Jan 8;14:1475114. doi: 10.3389/fcimb.2024.1475114 (PMC11750795; doi:10.3389/fcimb.2024.1475114)
Supplement: Supplementary file 3 [file Table2.doc]

**Supplementary table 2:** Details of sequences used for *Colletotrichum* phylogenetic analyses

| **Taxa** | **Voucher/Strain** | **GenBank accession number** | | | | |
| --- | --- | --- | --- | --- | --- | --- |
|  |  | **ITS** | ***GAPDH*** | ***CHS*-1** | ***ACT*** | ***tub*2** |
| *Colletotrichum truncatum* | CBS 151.35^T^ | GU227862 | GU228254 | GU228352 | GU227960 | GU228156 |
| *C. truncatum* | CBS 120709 | GU227877 | GU228269 | GU228367 | GU227975 | GU228171 |
| *C. truncatum* | CBS 141.79 | GU227873 | GU228265 | GU228363 | GU227971 | GU228167 |
| *C. truncatum* | IMI 135524 | GU227874 | GU228266 | GU228364 | GU227972 | GU228168 |
| *C. truncatum* | CBS 710.70 | GU227864 | GU228256 | GU228354 | GU227962 | GU228158 |
| *C. truncatum* | MFLUCC 22-0110 | OP740247 | OP744517 | OP744516 | OP744515 | OP744518 |
| *C. curcumae* | IMI 288937T | GU227893 | GU228285 | GU228383 | GU227991 | GU228187 |
| *C. acidi* | MFLUCC 17-2659^T^ | MG996505 | MH003691 | MH003694 | MH003697 | MH003700 |
| *C. subacidae* | CGMCC 3.20529^T^ | MZ595846 | OQ383226 | MZ799307 | OQ383224 | OQ470730 |
| *C. fusiforme* | MFLU 13- 0291^T^ | KT290266 | KT290255 | KT290253 | KT290251 | KT290256 |
| *C. aenigma* | ICMP 18608^T^ | JX010244 | JX010044 | JX009774 | JX009443 | JX010389 |
| *C. aeschynomenes* | ATCC 201874^T^ | JX010176 | JX009930 | JX009799 | JX009483 | JX010392 |
| *C. alienum* | ICMP 12071^T^ | JX010251 | JX010028 | JX009882 | JX009572 | JX010411 |
| *C. artocarpicola* | MFLUCC 18-1167^T^ | MN415991 | MN435568 | MN435569 | MN435570 | MN435567 |
| *C. asianum* | CBS 130418^T^ | JX010196 | JX010053 | JX009867 | JX009584 | JX010406 |
| *C. australianum* | BRIP 63698^T^ | KU923680 | MN442116 | MW092001 | MN442106 | KU923696 |
| *C. boninense* | CBS 123755^T^ | JQ005153 | JQ005240 | JQ005327 | JQ005501 | JQ005588 |
| *C. chamaedoreae* | LC:13868^T^ | MZ595890 | MZ664084 | MZ799274 | MZ664188 | MZ674008 |
| *C. chrysophilum* | CMM4268^T^ | KX094252 | KX094183 | KX094083 | KX093982 | KX094285 |
| *C. fructicola* | C1315.3^T^ | JX010165 | JX010033 | JX009866 | FJ907426 | JX010405 |
| *C. gloeosporioides* | CBS 112999^T^ | JQ005152 | JQ005239 | JQ005326 | JQ005500 | JQ005587 |
| *C. grossum* | CGMCC3.1 7614^T^ | KP890165 | KP890159 | KP890153 | KP890141 | KP890171 |
| *C. hystricis* | CBS 142411^T^ | KY856450 | KY856274 | KY856190 | KY856023 | KY856532 |
| *C. jiangxiense* | CGMCC 3.17363^T^ | KJ955201 | KJ954902 | OR826295 | KJ954471 | KJ955348 |
| *C. makassarense* | CPC:28612^T^ | MH728812 | MH728820 | MH805850 | MH781480 | MH846563 |
| *C. perseae* | CBS 141365^T^ | KX620308 | KX620242 | MZ799260 | KX620145 | KX620341 |
| *C. queenslandicum* | ICMP 1778^T^ | JX010276 | JX009934 | JX009899 | JX009447 | JX010414 |
| *C. salsolae* | ICMP 19051^T^ | JX010242 | JX009916 | JX009863 | JX009562 | JX010403 |
| *C. siamense* | CBS 130417 | FJ972613 | FJ972575 | JX009865 | FJ907423 | FJ907438 |
| *C. siamense* | CBS 125378^T^ | JX010278 | JX010019 | JX009875 | JX009441 | JX010410 |
| *C. siamense* | PHS005 | OR803029 | OR813955 | OL961570 | OR813959 | OR813963 |
| *C. siamense* | PHS006 | OR803030 | OR813956 | OR643684 | OR813960 | OR813964 |
| ***C. siamense*** | **RUFCC2455** | **PP989215** | **PQ014240** | **PQ014237** | **PQ014233** | **PQ014246** |
| ***C. siamense*** | **RUFCC2457** | **PP989216** | **PQ014241** | **PQ014238** | **PQ014234** | **PQ014247** |
| *C. siamense* | PHS009 | OR803032 | OR813957 | OR192993 | OR813961 | OR813965 |
| *C. tainanense* | CBS 143666^T^ | NR_171185 | MH728823 | MH805857 | MH781475 | MH846558 |
| ***C. truncatum*** | **RUFCC2451** | **PP989217** | **PQ014242** | **PQ014239** | **PQ014235** | **PQ014248** |
| *C. wuxiense* | CGMCC 3.17894^T^ | KU251591 | KU252045 | KU251939 | KU251672 | KU252200 |
| *C. xanthorrhoeae* | ICMP 17903^T^ | JX010261 | JX009927 | JX009823 | JX009478 | JX010448 |
