# Supplementary material for: Ectophoma salviniae sp. nov., Neottiosporina mihintaleensis sp. nov. and four other endophytes associated with aquatic plants from Sri Lanka and their extracellular enzymatic potential
Source: Front Cell Infect Microbiol. 2025 Jan 8;14:1475114. doi: 10.3389/fcimb.2024.1475114 (PMC11750795; doi:10.3389/fcimb.2024.1475114)
Supplement: Supplementary file 4 [file Table3.doc]

**Supplementary table 3:** Details of sequences used for *Ectophoma* phylogenetic analyses

| **Taxa** | **Voucher/Strain** | **GenBank accession number** | | | |
| --- | --- | --- | --- | --- | --- |
|  |  | **ITS** | **LSU** | ***rpb*2** | ***tub*2** |
| *Ectophoma insulana* | CBS 252.92^T^ | MN973481 | MN943685 | MT018070 | MT005581 |
| *E. multirostrata* | CBS 274.60^T^ | FJ427031 | GU238111 | LT623265 | FJ427141 |
| *E. multirostrata* | CBS 368.65 | FJ427033 | GU238112 | N/A | FJ427143 |
| *E. iranica* | SCUA-K1G1^T^ | MK519382 | MK519389 | N/A | MK519562 |
| *E. iranica* | SCUA-K1 | MK519381 | MK519388 | N/A | MK519561 |
| *E. myriophyllanus* | YMF1.05050^T^ | MH257417 | MH257502 | MH311848 | MH423013 |
| *E. myriophyllanus* | YMF1.05208 | MH257418 | MH257503 | MH311849 | MH423014 |
| *E. phoenicis* | ZHKUCC 22-0163^T^ | OQ275208 | OQ275194 | OQ343375 | OQ336259 |
| *E. phoenicis* | ZHKUCC 22-0164 | OQ275209 | OQ275195 | OQ343376 | OQ336260 |
| *E. pomi* | CBS 267.92^T^ | N/A | GU238128 | LT623263 | GU237643 |
| *E. pomi* | CBS 121.93 | MN972933 | MN973320 | MN983570 | MN983948 |
| ***E. salviniae*** | **RUFCC2458^T^** | **PP989218** | **PP989224** | **PQ014244** | N/A |
| ***E. salviniae*** | **RUFCC2462** | **PP989219** | **PP989225** | **PQ014245** | N/A |
| *Didymella exigua* | CBS 183.55^T^ | GU237794 | EU754155 | EU874850 | GU237525 |
