# Supplementary material for: Ectophoma salviniae sp. nov., Neottiosporina mihintaleensis sp. nov. and four other endophytes associated with aquatic plants from Sri Lanka and their extracellular enzymatic potential
Source: Front Cell Infect Microbiol. 2025 Jan 8;14:1475114. doi: 10.3389/fcimb.2024.1475114 (PMC11750795; doi:10.3389/fcimb.2024.1475114)
Supplement: Supplementary file 5 [file Table4.doc]

**Supplementary table 4:** Details of sequences used for *Neottiosporina* phylogenetic analyses

| **Taxa** | **Voucher/Strain** | **GenBank accession number** | | |
| --- | --- | --- | --- | --- |
|  |  | **ITS** | **LSU** | **SSU** |
| *Neottiosporina cylindrica* | BRIP 16231 | MZ734405 | N/A | N/A |
| *N. cylindrical* | BRIP 14187^T^ | MZ734404 | N/A | N/A |
| ***N. mihintaleensis*** | **RUFCC2454^T^** | **PP989220** | **PP989226** | **PP989229** |
| ***N. mihintaleensis*** | **RUFCC2461** | **PP989221** | **PP989227** | **PP989230** |
| *N. paspali* | CBS 33 1.37 | KP170653 | N/A | N/A |
| *Helminthosporiella stilbacea* | COAD 2126 | MG668862 | N/A | N/A |
| *Semifissispora tooloomensis* | CBS 143431^T^ | NR_156674 | N/A | N/A |
| *S. rotundata* | CPC 549^T^ | KT950847 | N/A | N/A |
| *S. natalis* | CPC 25383^T^ | NR_145195 | N/A | N/A |
| *Suttonomyces rosae* | MFLUCC 15-0051^T^ | NR_157548 | N/A | N/A |
