# Supplementary material for: Ectophoma salviniae sp. nov., Neottiosporina mihintaleensis sp. nov. and four other endophytes associated with aquatic plants from Sri Lanka and their extracellular enzymatic potential
Source: Front Cell Infect Microbiol. 2025 Jan 8;14:1475114. doi: 10.3389/fcimb.2024.1475114 (PMC11750795; doi:10.3389/fcimb.2024.1475114)
Supplement: Supplementary file 6 [file Table5.doc]

**Supplementary table 5:** Details of sequences used for *Phyllosticta capitalensis* phylogenetic analyses

| **Taxa** | **Voucher/Strain** | **GenBank accession number** | | | |
| --- | --- | --- | --- | --- | --- |
|  |  | **ITS** | ***tef*1** | ***ACT*** | ***GADPH*** |
| *Phyllosticta acaciigena* | CPC 28295^T^ | KY173433 | N/A | KY173570 | N/A |
| *P. aloeicola* | CPC 21020^T^ | KF154280 | KF289193 | KF289311 | KF289124 |
| *P. ardisiicola* | NBRC 102261^T^ | AB454274 | N/A | AB704216 | N/A |
| *P. aristolochiicola* | BRIP 53316^T^ | JX486129 | N/A | N/A | N/A |
| *P. azevinhi* | MUCC0088 | AB454302 | N/A | AB704226 | N/A |
| *P. beaumarisii* | CBS 535.87 | NR_145235 | KF766429 | KF306232 | KF289074 |
| *P. brazilianiae* | LGMF 330^T^ | JF343572 | JF343593 | JF343656 | JF343758 |
| *P. capitalensis* | CBS 128856^T^ | JF261465 | JF261507 | JF343647 | JF343776 |
| *P. capitalensis* | CBS 114751 | EU167584 | FJ538407 | FJ538465 | KF289088 |
| ***P. capitalensis*** | **RUFCC2452** | **PP989222** | **PQ014249** | **PQ014236** | **PQ014243** |
| *P. carochlae* | CGMCC 3.17317^T^ | KJ847422 | KF289178 | KF289273 | KF289092 |
| *P. cavendishii* | BRIP 57384 | KC117644 | KF009695 | KF014059 | KU716085 |
| *P. cordylinophila* | MFLUCC 10-0166^T^ | KF170287 | KF289172 | KF289295 | KF289076 |
| *P. doitungensis* | MFLU 21-0175 T | OK661033 | OL345581 | N/A | N/A |
| *P. eugeniae* | CBS 445.82 T | AY042926 | KF289208 | KF289246 | KF289139 |
| *P. fallopiae* | MUCC0113 T | AB454307 | N/A | AB704228 | N/A |
| *P. guangdongensis* | CFCC 58144 T | OQ202160 | OQ267758 | OQ267764 | OQ267770 |
| *P. ilicis-aquifolii* | CGMCC 3.14358 T | JN692538 | JN692526 | JN692514 | N/A |
| *P. maculate* | CPC 18347 T | JQ743570 | KF009700 | KF014016 | N/A |
| *P. mangiferae* | IMI 260576 T | JF261459 | JF261501 | JF343641 | JF343748 |
| *P. mangifera-indicae* | MFLUCC 10-0029 T | KF170305 | KF289190 | KF289296 | KF289121 |
| *P. musaechinensis* | GZAAS 6.1247 | KF955294 | KM816639 | KM816627 | KM816633 |
| *P. musaechinensis* | GZAAS 6.1384 | KF955295 | KM816640 | KM816628 | KM816634 |
| *P. musarum* | BRIP 57803 | JX997138 | KF009737 | KF014055 | N/A |
| *P. oblongifoliae* | SAUCC210052 T | OM248445 | OM273893 | OM273897 | OM273901 |
| *P. paracapitalensis* | CPC 26517 T | KY855622 | KY855951 | KY855677 | KY855735 |
| *P. parthenocissi* | CBS 111645 T | EU683672 | JN692530 | JN692518 | N/A |
| *P. partricuspidatae* | NBRC 9466 T | KJ847424 | KJ847446 | KJ847432 | KJ847440 |
| *P. philoprina* | CBS 587.69 | KF154278 | KF289206 | KF289250 | KF289137 |
| *P. phoenicis* | CBS 147091 | MW883442 | MW890098 | MW890031 | MW890050 |
| *P. pterospermi* | SAUCC210104 T | OM249954 | OM273902 | OM273904 | OM273906 |
| *P. rhizophorae* | NCYUCC 19-0352 T | MT360030 | N/A | MT363248 | MT363250 |
| *P. schimae* | CGMCC 3.14354 T | JN692534 | JN692522 | JN692510 | JN692506 |
| *P. schimicola* | CGMCC 3.17319T | KJ847426 | KJ847448 | KJ847434 | KJ854895 |
| *P. styracicola* | CGMCC3.14985 T | JX025040 | JX025045 | JX025035 | JX025030 |
| *P. vitis-rotundifoliae* | CGMCC 3.17322 T | KJ847428 | KJ847450 | KJ847436 | KJ847442 |
| *Botryosphaeria obtusa* | CMW 8232 T | AY972105 | DQ280419 | AY972111 | N/A |
| *B. stevensii* | CBS 112553 T | AY259093 | AY573219 | N/A | N/A |
